# Synthesis of HBC fluorophores with an electrophilic handle for covalent attachment to Pepper RNA

**DOI:** 10.3762/bjoc.21.56

**Published:** 2025-04-04

**Authors:** Raphael Bereiter, Ronald Micura

**Affiliations:** 1 Institute of Organic Chemistry, Center for Molecular Biosciences, Innsbruck (CMBI), University of Innsbruck, Innrain 80-82, 6020 Innsbruck, Austria

**Keywords:** covalent RNA labeling, FLAP, fluorophore synthesis, HBC530, self-alkylating ribozymes

## Abstract

The fluorescent light-up aptamer (FLAP) Pepper can utilize fluorophores that are equipped with an electrophilic handle for the covalent attachment of the surrogate to the RNA. The resulting irreversibly tethered dye–RNA complexes have opened up new avenues for RNA imaging in live cells. Here, we report the syntheses of such modified HBC530 ((4-((2-hydroxyethyl)(methyl)amino)benzylidene)cyanophenylacetonitrile) fluorophores for easy access, which will contribute to the rapid dissemination of the RNA imaging approaches associated therewith.

## Introduction

The discovery of fluorescent reporters, such as green fluorescent protein (GFP), has revolutionized genetics by providing highly accurate real-time detection of fusion proteins in vitro and in vivo [1]. Pioneering work on GFP-tagged proteins for real-time monitoring of gene expression was first reported by Chalfie and co-workers in 1994 [2]. For a long time, however, there was no complementary RNA-based tool with comparable live-cell-imaging properties. Based on the autocatalytically generated fluorophore 4-(*p*-hydroxybenzylidene)imidazolidin-5-one (HBI) in GFP, in vitro selection on derivatives of HBI led to the discovery of the first fluorogen-activating aptamer (FLAP) in the family of “RNA mimics of GFP”, called Spinach [3]. This aptamer was used to study intracellular RNA dynamics in living cells and was the starting point for a series of in vitro selected FLAPs, e.g., Corn [4], Chili [5], Mango [6], Pepper [7], Clivia [8], and Okra [9]. All known FLAPs are non-covalently bound to their fluorophore and despite high (usually nanomolar) binding affinities this can pose problems for RNA live-cell imaging. For instance, diffusion of the ligand and non-specific binding lead to unfavorable background and/or signal loss [10].

**Figure 1:**
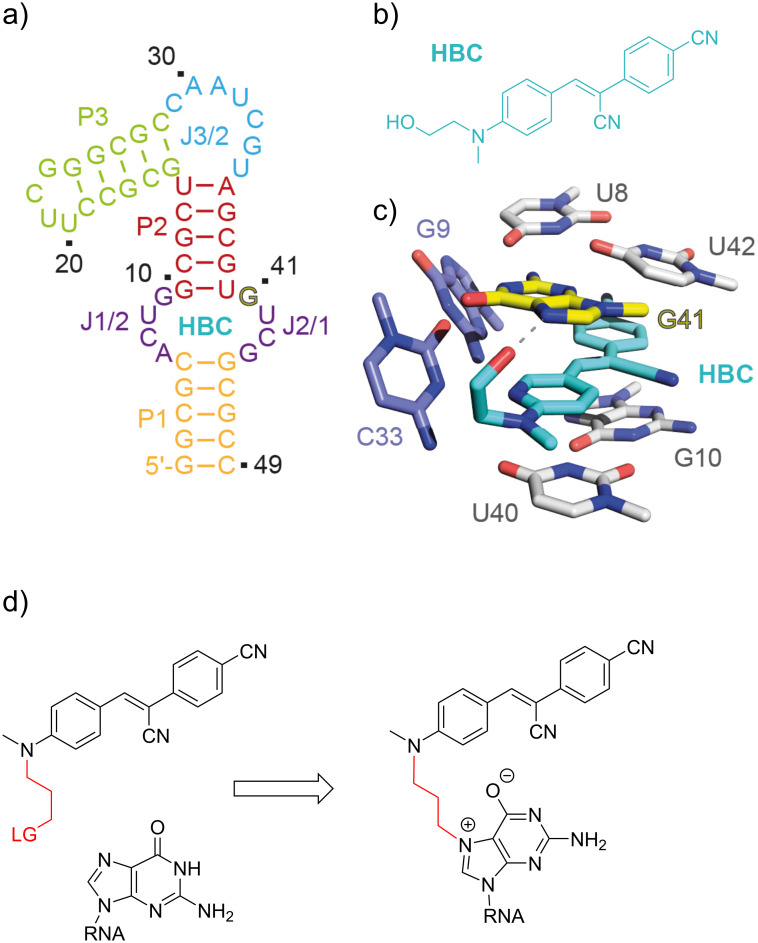
Structure-guided approach for engineering the (non-covalent) fluorescent light-up aptamer Pepper into its covalent counterpart [11]. a) Secondary structure of the Pepper aptamer [12]; b) chemical structure of the fluorophore HBC530 [7]; c) three-dimensional structure of the Pepper binding site with a bound HBC derivative (pdb code 7EOM). The hydrogen bond between N7 of guanine in position 41 (G41) and the hydroxy group of HBC is highlighted as gray dashed line [12]; d) concept for covalent attachment of HBC fluorophores to the N7 atom of G41 of the RNA. Key is the functionalization of the original *N*-(2-hydroxyethyl) moiety into a reactive handle providing a mild electrophile (LG), such as, e.g., *N*-(3-bromopropyl) or *N*-(3-mesyloxypropyl) [11].

To address these problems, our research group has recently developed the first covalent FLAP system (*co*FLAP) based on the Pepper aptamer and demonstrated some advantages of the covalent (*co*Pepper) over the non-covalent (Pepper) reporter in RNA imaging (Figure 1) [11]. Here, we describe the full synthesis of “self-attaching” Pepper fluorophores that provide the parent HBC530 ((4-((2-hydroxyethyl)(methyl)amino)benzylidene)cyanophenylacetonitrile) stilbene core [7] but offer an electrophilic alkyl handle on the amino group replacing the original *N*-hydroxyethyl residue. Three of them have been applied in the cellular applications (Brc_3_HBC (**8**), MsOc_3_HBC (**14**), and MsOc_3_HBC-vinyl (**22**)) as reported previously [11]. Nine more HBC derivatives with altered handle lengths and different electrophilic groups are introduced in this study. Furthermore, we include an evaluation of the efficiency of all reactive fluorophores (previously introduced and the new ones) for covalent attachment to the Pepper aptamer in vitro.

## Results and Discussion

### Background

**Scheme 1:**
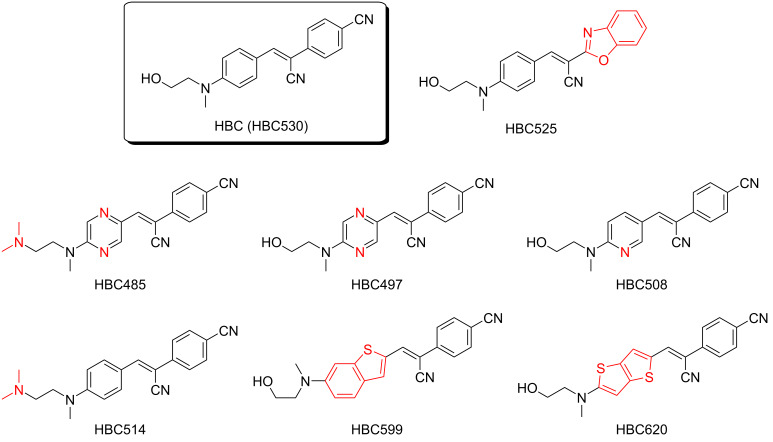
Chemical structures of the HBC dye family [7]. Variations to HBC530 highlighted in red color. All dyes shown bind non-covalently to Pepper RNA, a fluorescent light-up aptamer. For one of them (HBC530), we describe the synthesis of a series of derivatives/analogs that contain reactive handles (see main text and the figures below). In principle, the handles can also be applied at the other members of the HBC family, with the expectation of the same reactivity towards Pepper RNA.

The fluorescent light-up aptamer Pepper binds a series of structurally related synthetic dyes that contain a stilbene core. The lead compound is (4-((2-hydroxyethyl)(methyl)amino)benzylidene)cyanophenylacetonitrile, called HBC [7]. This typical push–pull fluorophore uses a dialkylamino group as the electron donor and a cyano group as the electron acceptor, while the connecting conjugating system has been varied to obtain a series of derivatives covering a broad spectral range (Scheme 1) [7,12]. All of these fluorophore derivatives bind the Pepper aptamer with affinities in the low nanomolar range [12]. Crystal structure analyses revealed that in the ligand binding pocket, a characteristic hydrogen bond is formed between the hydroxy group of the *N*-hydroxyethyl substituent of HBC and the N7 of G41 [12–13]. We have shown that this feature can be exploited to construct a covalent bond between the fluorophore and the RNA by replacing the *N*-hydroxyethyl group of the dye with an electrophilic handle, resulting in efficient RNA alkylation at the N7 of G41 [11].

### Synthesis and evaluation of Pepper dyes with an electrophilic handle

**Scheme 2:**
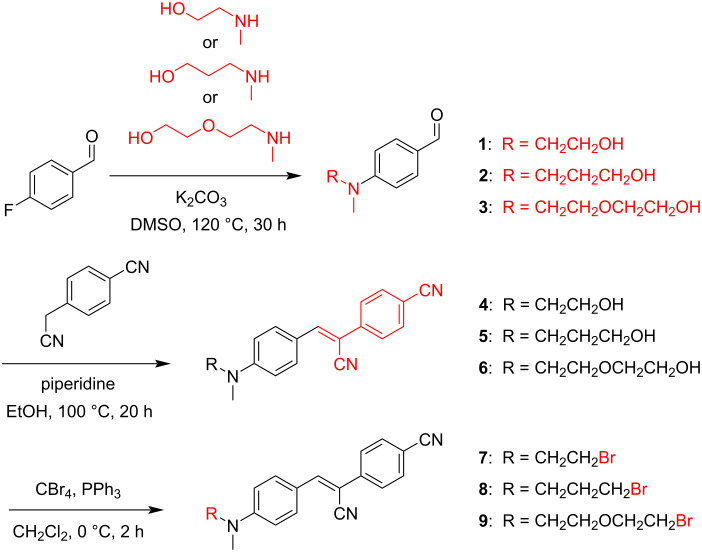
Synthesis of bromoalkyl HBC derivatives **7**, **8**, and **9**.

In analogy to the reported synthesis of HBC [7], we first developed a route to generate a series of HBC derivatives with *N*-(bromoalkyl) handles of different lengths (Scheme 2), with the intention of optimizing the RNA alkylation reaction for high yields. The synthesis starts with a nucleophilic aromatic substitution of 4-fluorobenzaldehyde with 2-(methylamino)ethanol, 3-methylamino-1-propanol or 2-[2-(methylamino)ethoxy]ethan-1-ol in the presence of potassium carbonate to afford benzaldehyde derivatives **1**, **2**, and **3** in excellent yields. Next, the piperidine-induced condensation with 4-cyanophenylacetonitrile afforded HBC **4** and HBC-like ligands **5** and **6** as bright orange solids. Finally, the bromo group was introduced under Appel conditions with carbon tetrabromide and triphenylphosphine to give the fluorophores **7, 8**, and **9**, with linker lengths of two, three, and five atoms.

**Scheme 3:**
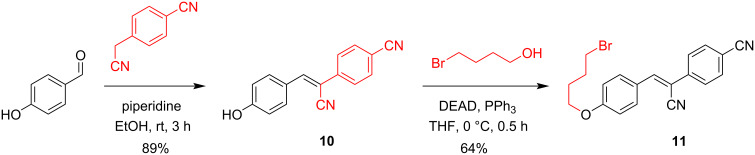
Synthesis of the HBC ether derivative **11**.

Unfortunately, this strategy was impractical for the HBC derivative with a C4-handle (*N*-(4-bromobutyl)-HBC) due to intramolecular cyclization with the amine. To prevent intramolecular cyclization, we considered the 4-bromobutyl HBC ether **11** as a potential candidate with a 4-atom spacer. Accordingly, 4-hydroxybenzaldehyde was reacted with 4-cyanophenylacetonitrile to give compound **10**, followed by installing the bromobutyl handle with 4-bromobutanol under Mitsunobu conditions (Scheme 3).

**Figure 2:**
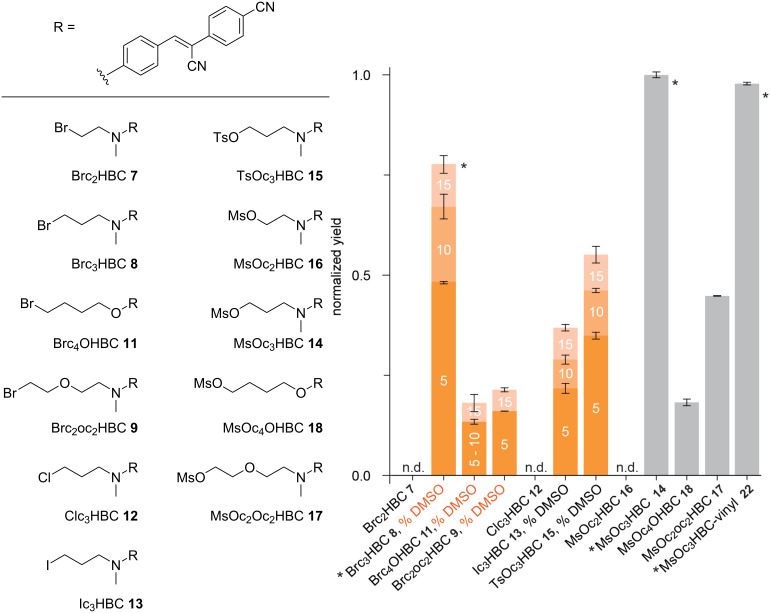
Pepper aptamer reacts with different HBC derivatives. Chemical structures of the HBC derivatives used (left side). Bar graph (right side) illustrating the relative yields of covalent tethering under the following conditions: 2.5 µM RNA, 50 µM HBC ligand, 100 mM KCl, 2 mM MgCl_2_, 50 mM HEPES buffer, pH 7.0, 5 h at 37 °C. The numbers in the orange bars indicate the percentage of DMSO (%) used as co-solvent; grey bars indicate that no co-solvent was used; n.d. not detected (neither in buffer nor in buffer/DMSO reaction solutions). For the chemical structure of compound **22** see Scheme 6. Measurements were performed in three independent experiments. The entries marked with asterisks indicate the fluorophores with the highest reactivity, they are consistent with ref. [11] and serve as references in this work.

Next, HBC dyes **7**, **8**, **9**, and **11** were tested for their reactivity with the Pepper aptamer. They were incubated together with the RNA in buffer containing potassium and magnesium ions at a physiological pH of 7.0 for 5 hours. Analysis of the reaction mixture by anion-exchange HPLC revealed that the bromopropyl handle (C3 homolog **8**) gave the highest yield of covalently tethered HBC-RNA complex (50%). Significantly less RNA alkylation yield was observed for the HBC ether **11** (C4 homolog) and the bromoethoxyethyl HBC **9** (“C5” homolog). No reaction was observed for the C2 homolog **7** (Figure 2). Notably, the reaction yields for all derivatives increased when the DMSO content of the reaction mixture was increased from 5 to 15%. (DMSO was initially used to avoid precipitation of the compounds **7**, **8**, **9**, and **11** during long (>12 h) incubation times).

**Scheme 4:**
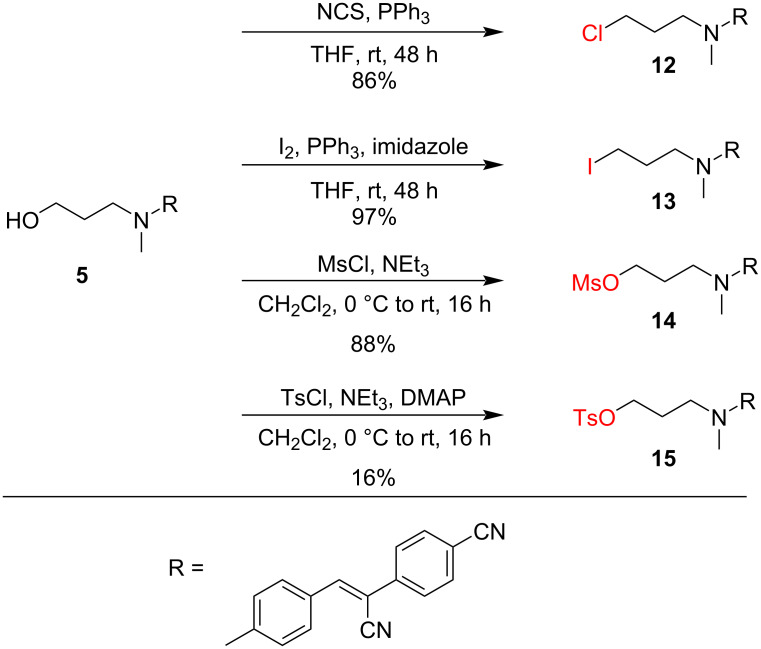
Derivatization of the HBC fluorophore **5** to generate handles with distinct electrophilic groups.

In view of these results, we further focused on HBC derivatives with the high-yielding C3 handle and sought a variation of the electrophile. Thus, adapted Appel reaction conditions using *N*-chlorosuccinimide and iodine, respectively, were applied to compound **5** to give the chloropropyl and iodopropyl HBC dyes **12** and **13** (Scheme 4). In addition, the HBC alcohol **5** was reacted under basic conditions with methanesulfonyl chloride to give the *N*-(3-mesyloxypropyl) HBC derivative **14**, or with tosyl chloride to give the *N*-(3-tosyloxypropyl) HBC derivative **15** (Scheme 4). It should be noted that attempts to prepare and isolate *N*-(3-trifluoromethansulfonylpropyl)-modified HBC failed, probably due to rapid hydrolysis during workup.

Next, the HBC dyes **12**, **13**, **14**, and **15** were tested for their reactivity with the Pepper RNA aptamer, using the same conditions as described above for the first series of RNA alkylation experiments (Figure 2). Analysis by HPLC revealed that the chloropropyl HBC derivative **12** was not reactive. Surprisingly, the iodopropyl HBC derivative **13** gave significantly lower yields than its bromo counterpart. The tosyloxypropyl HBC analog **15** was also less reactive than its bromo counterpart. Of all the HBC dyes with reactive handles synthesized to date, the highest RNA alkylation yield was obtained with the *N*-(3-mesyloxypropyl) HBC derivative **14**. In addition, the mesyl compound **14** showed significantly better solubility in the reaction buffer and did not require DMSO as co-solvent, making it a very promising candidate for in vivo applications.

**Scheme 5:**
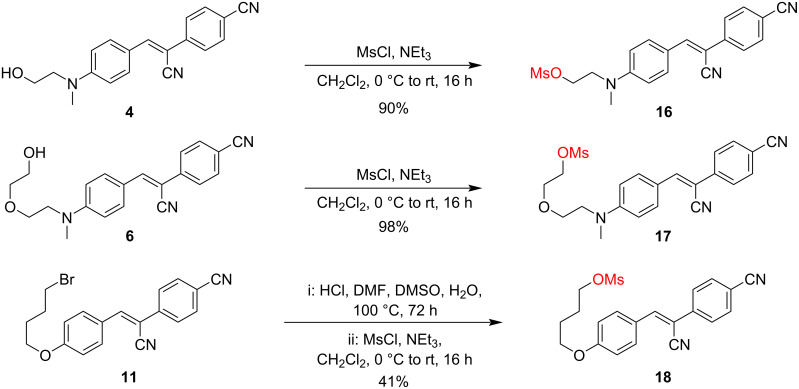
Synthesis of mesylated HBC fluorophores **16**, **17**, and **18**.

To complete the study, and given that the mesyloxy group has shown superior efficacy over all other functional groups tested for Pepper alkylation, we additionally synthesized the HBC series with different linker lengths with the mesyloxy group, yielding the derivatives **16** to **18** (Scheme 5). All of these derivatives – although well soluble in the reaction buffer without the need for additional DMSO – were less reactive than *N*-(3-mesyloxypropyl) HBC derivative **14** (Figure 2).

### Synthesis of a bifunctional Pepper dye

**Scheme 6:**
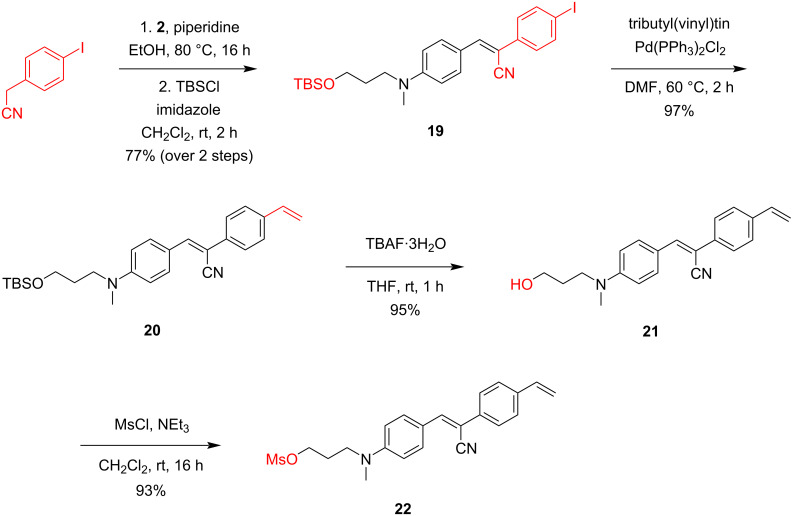
Synthesis of the bifunctional HBC fluorophore **22**. For an application of **22** (pulldown of circular Pepper RNA from total RNA of HEK 293T cells) see reference [11].

Encouraged by the efficient attachment of the bromo- and mesyloxypropyl-modified HBC fluorophores to the Pepper aptamer, we generated a bifunctional HBC ligand with a second handle that is available for bioorthogonal reactions. The original intention was to provide an RNA pulldown tool where the RNA of interest tagged with the Pepper aptamer becomes covalently attached to the fluorophore, which in turn can be biotinylated via a catalyst-free inverse-electron-demand Diels–Alder reaction (IEDDA) [11]. Thereby, the entire process is easily monitored by the inherent fluorescent signal of the target RNA [11]. The synthetic route to such an HBC fluorophore is shown in Scheme 6. Piperidine-induced condensation of compound **2** with 4-iodophenylacetonitrile afforded the HBC-like ligand, whose hydroxy group was immediately protected with TBS-Cl to provide fluorophore **19**. Subsequent palladium-catalyzed cross-coupling with tributyl(vinyl)tin resulted in the installation of the vinyl group (compound **20**). Finally, cleavage of the silyl ether gave the free alcohol **21**, which was converted into the corresponding mesyloxypropyl HBC ligand **22**. The usefulness of this type of bifunctional fluorescent ligands in RNA affinity purification has recently been demonstrated by our group [11].

## Conclusion

Covalent fluorescent light-up aptamers (*co*FLAPs) are opening new avenues for RNA imaging [11]. In this work, we describe robust synthetic routes for twelve HBC fluorophores with an electrophilic handle, three of which have been previously used in cellular applications [11]. Comparative reactivity analysis of all these fluorophores revealed that the *N*-(3-mesyloxypropyl) HBC **14** is the most efficient for covalent tethering to Pepper RNA. Moreover, the mesyloxyalkyl fluorophores also exhibited favorable solubility compared to the other functionalizations which is a critical aspect for applications in the cell.

We also note that primary alkyl halides, and in particular, the mesyloxy alkyl handles presented here, are relatively underexplored for covalent labeling of nucleic acids. Rather, halides with enhanced electrophilic potency, such as α-halocarbonyls [14–16], nitrogen (half-)mustards [17–18], or epoxides [19–20], Michael acceptors [17], carbamates [17], imidazolides [17], squarates [17], and diaziridines [17] (the latter requiring photoactivation) have been used, although often with limited success. We believe that the electrophilic warheads presented here offer an excellent balance between reactivity and selectivity for labeling of nucleic acids, and therefore, may also stimulate new designs for RNA targeting and RNA drugging.

## Experimental

**General.** Chemical reagents and solvents were purchased in the highest available quality from commercial suppliers (Merck/Sigma-Aldrich, ABCR) and used without further purification. Analytical thin-layer chromatography (TLC) was performed on Macherey-Nagel Polygram^®^ SIL G/UV254 plates. 0.2 mm silica gel 60 for column chromatography was purchased from Macherey-Nagel. ^1^H and ^13^C NMR spectra were recorded on a Bruker UltrashieldTM 400 MHz Plus or a 700 MHz Avance Neo spectrometer. Chemical shifts (δ) are reported relative to tetramethylsilane (TMS), referenced to the residual solvent signal (DMSO-*d*_6_: 2.50 ppm for ^1^H NMR and 39.52 ppm for ^13^C NMR spectra; CDCl_3_: 7.26 ppm for ^1^H NMR and 77.16 ppm for ^13^C NMR spectra). Signal assignments are based on ^1^H-^1^H-COSY, ^1^H-^13^C-HSQC and ^1^H-^13^C-HMBC experiments. High-resolution mass spectra were recorded in positive ion mode unless otherwise noted on a Thermo Scientific Q Exactive Orbitrap.

**General procedure A.** 4-Fluorobenzaldehyde, the corresponding *N*-methylated amino alcohol and potassium carbonate were suspended in dimethyl sulfoxide and stirred for 30 hours at 120 °C. The resulting suspension was poured on crushed ice and extracted four times with chloroform, dried over Na_2_SO_4_ and concentrated to dryness. Finally, the crude compound was purified via silica gel chromatography using 50–70% ethyl acetate in cyclohexane as gradient.

**General procedure B.** The product obtained in general procedure A was dissolved in ethanol mixed with 4-cyanophenylacetonitrile and 8 drops of piperidine and stirred at 100 °C for 20 hours. A strongly yellow-colored solution was obtained and cooled on ice, whereby a precipitate was formed and filtered off. The filter cake was washed with ice-cold ethanol and dried under high vacuum.

**General procedure C.** In a manner similar to [11], the product obtained in general procedure B was dissolved in dichloromethane and cooled to 0 °C under argon atmosphere. Then, triphenylphosphine and carbon tetrabromide were added and stirred at room temperature for two hours. Afterwards, the entire mixture was loaded on a silica gel column and eluted with 100% dichloromethane.

**General procedure D.** In a manner similar to [11], the product obtained in general procedure B, methanesulfonyl chloride, and triethylamine were dissolved in dichloromethane and stirred overnight at room temperature. After reaction control and 100% consumption of the starting material, the entire mixture was poured on a silica gel column and the product was eluted using 0–1% methanol in dichloromethane.

**In vitro reaction of BrC_3_ or MsOC_3_ ligands with Pepper RNA.** In a manner analogous to [11], a typical alkylation reaction was carried out in a volume of 60 µL. Pepper RNA (0.15 nmol) was dissolved in 40 µL of water, followed by the addition of 12 µL of buffer (250 mM HEPES, 500 mM KCl, pH 7.0) and 6.0 µL of MgCl_2_ solution (20 mM). The aptamer was annealed by heat shock at 90 °C for 2 minutes and cooled on ice. Then, 2.0 µL of a ligand stock solution (3.0 mM, in DMSO) was added. The final concentrations of the reaction mixture were: 2.5 µM RNA, 50 µM ligand, 50 mM HEPES, 100 mM KCl and 2.0 mM MgCl_2_. After incubation (37 °C, 5 hours), the reaction was quenched by adding 40 µL of a Na_2_H_2_EDTA solution (200 mM) to reach a final concentration of 80 mM Na_2_H_2_EDTA in a volume of 100 µL. Each sample was analyzed by AE chromatography (Dionex DNAPac PA-100 column; 4 mm × 250 mm) at 80 °C with a flow rate of 1.0 mL/min. A gradient of 25–37.5% B in A in 25.0 minutes was used; Eluent A: 25 mM Tris∙HCl, 10 mM NaClO_4_, 20% acetonitrile, pH 8.0; eluent B: 25 mM Tris∙HCl, 600 mM NaClO_4_, 20% acetonitrile, pH 8.0. HPLC traces were recorded with UV absorption by 260 nm.

## Supporting Information

File 1 Experimental, characterization data and copies of NMR spectra.

## Data Availability

All data that supports the findings of this study is available in the published article and/or the supporting information of this article.
