# Supplementary material for: Synthesis of HBC fluorophores with an electrophilic handle for covalent attachment to Pepper RNA
Source: Beilstein J Org Chem. 2025 Apr 4;21:727–35. doi: 10.3762/bjoc.21.56 (PMC11995720; doi:10.3762/bjoc.21.56)

BEILSTEIN JOURNAL OF ORGANIC CHEMISTRY

Supporting Information

for

Synthesis of HBC fluorophores with an electrophilic handle for covalent attachment to Pepper RNA

Raphael Bereiter and Ronald Micura

Beilstein J. Org. Chem. **2025,** *21,* 727–735. doi:10.3762/bjoc.21.56

Experimental, characterization data and copies of NMR spectra

License and Terms: This is a supporting information file under the terms of the Creative Commons Attribution License (https://creativecommons.org/licenses/by/4.0). Please note that the reuse, redistribution and reproduction in particular requires that the author(s) and source are credited and that individual graphics may be subject to special legal provisions.
The license is subject to the *Beilstein Journal of Organic Chemistry* terms and conditions: (https://www.beilstein-journals.org/bjoc/terms)

Table of contents

Synthesis and characterization data S2

References S10

NMR spectra of compounds **1–7, 9**–**13**, and **15**–**21** S12

4-[(2-Hydroxyethyl)(methyl)amino]benzaldehyde (1)

Compound **1** was synthesized following general procedure A using 4-fluorobenzaldehyde (528 mg, 4.25 mmol), 2-(methylamino)ethanol (479 mg, 513 µL, 6.38 mmol) and potassium carbonate (K_2CO_3, 741 mg, 5.40 mmol) in 3.7 mL dimethyl sulfoxide. Yield: 573 mg of compound **1** as a white solid (75%). TLC: (CH_2Cl_2 / methanol, 9:1): R_f = 0.6. HR-ESI-MS (m/z): $[M+H]^+$ calcd.: 180.1024; found: 180.1002. ^1H NMR: (400 MHz, DMSO-d_6, 25 °C): δ = 3.05 (3H, s, ***H₃***C), 3.50 – 3.60 (4H, m, -$\boldsymbol{H_2}$C-$\boldsymbol{H_2}$C-), 4.76 (1H, t, *J* = 5.35 Hz,-O***H***), 6.79 (2H, d, *J* = 8.95 Hz, -C***H***(meta)), 7.64 (2H, d, *J* = 8.99 Hz, -C***H***(ortho)), 9.65 (1H, s, C***H***O). ^{13}C NMR: (100 MHz, DMSO-d_6, 25 °C): δ = 38.84 (***C***H_3), 53.84 (O-H_2CH_2***C***-N), 58.07 (O-H_2***C***H_2C-N), 110.94 (***C***H(meta)), 124.31 (***C***-NR_2), 131.58 (***C***H(ortho)), 153.57 (***C***-CHO), 189.65 (***C***HO).

4-[(3-Hydroxypropyl)(methyl)amino]benzaldehyde (2)

Compound **2** was synthesized following general procedure A using 4-fluorobenzaldehyde (495 mg, 4.00 mmol), 3-methylamino-1-propanol (533 mg, 582 µL, 6.00 mmol) and potassium carbonate (K_2CO_3, 695 mg, 5.00 mmol) in 3.5 mL dimethyl sulfoxide. Yield: 577 mg of compound **2** as a yellowish oil (75%). TLC: (CH_2Cl_2 / methanol, 9:1): R_f = 0.6. HR-ESI-MS (m/z): $[M+H]^+$ calcd.: 194.1181, found: 194.1241. ^1H NMR: (400 MHz, DMSO-d_6, 25 °C): δ = 1.69 (2H, p, *J* = 6.67 Hz, -CH_2C$\boldsymbol{H_2}$$CH_2$-), 3.01 (3H, s, $\boldsymbol{H_3}$C), 3.43 – 3.51 (4H, m, -C$\boldsymbol{H_2}$$CH_2C\boldsymbol{H_2}$-), 4.57 (1H, t, *J* = 4.96 Hz,-O***H***), 6.79 (2H, d, *J* = 8.94 Hz, -C***H***(meta)), 7.65 (2H, d, *J* = 8.97 Hz, -C***H***(ortho)), 9.65 (1H, s, C***H***O). ^{13}C NMR: (100 MHz, DMSO-d_6, 25 °C): δ = 30.02 (O-CH_2***C***H_2CH_2-N), 38.51 (***C***H_3), 49.03 (O-CH_2CH_2***C***H_2-N), 58.60 (O-***C***$H_2CH_2CH_2$-N), 111.33 (***C***H(meta)), 124.74 (***C***-NR_2), 132.12 (***C***H(ortho)), 153.80 (***C***-CHO), 190.12 (***C***HO).

4-[2-(2-Hydroxyethoxy)ethyl(methyl)amino]benzaldehyde (3)

Compound **3** was synthesized following general procedure A using 4-fluorobenzaldehyde (420 mg, 3.40 mmol), 2-[2-(methylamino)ethoxy]ethan-1-ol (605 mg, 5.10 mmol) and potassium carbonate (K_2CO_3, 589 mg, 4.30 mmol) in 3.0 mL dimethyl sulfoxide. Yield: 642 mg of compound **3** as a yellow oil (85%). TLC: (CH_2Cl_2 / methanol, 9:1): R_f = 0.6. HR-ESI-MS (m/z): $[M+H]^+$ calcd.: 224.1287, found: 224.1302. ^1H NMR: (400 MHz, DMSO-d_6, 25 °C): δ = 3.04 (3H, s, $\boldsymbol{H_3}$C), 3.43 (2H, m, (HO-CH_2C$\boldsymbol{H_2}$-O)), 3.47 (2H, m, (HO-C$\boldsymbol{H_2}$$CH_2$-O)), 3.60 (4H, m, (O-(C$\boldsymbol{H_2}$$)_2$-N)), 4.57 (1H, t, *J* = 5.37 Hz,-O***H***), 6.81 (2H, d, *J* = 8.95 Hz, -C***H***(meta)), 7.66 (2H, d, *J* = 9.02 Hz, -C***H***(ortho)), 9.66 (1H, s, C***H***O). ^{13}C NMR: (100 MHz, DMSO-d_6, 25 °C): δ = 38.77 (***C***H_3), 51.13 (O-CH_2***C***H_2-N), 60.25 (HO-CH_2***C***H_2-N), 67.64 (O-***C***H_2CH_2-N), 72.40 (O-CH_2***C***H_2-N), 111.05 (***C***H(meta)), 124.51 (***C***-NR_2), 131.61 (***C***H(ortho)), 153.41 (***C***-CHO), 189.75 (***C***HO).

[4-((2-Hydroxyethyl)(methyl)amino)-benzylidene]-4-cyanophenylacetonitrile (4) [1]

Compound **4** was synthesized following general procedure B using compound **1** (422 mg, 2.40 mmol) and 4-cyanophenylacetonitrile (352 mg, 2.50 mmol) in 40 mL ethanol. Yield: 583 mg of compound **4** as an orange solid (82%). HR-ESI-MS (m/z): $[M+H]^+$ calcd.: 304.1449, found: 304.1432 ^1H NMR: (400 MHz, DMSO-*d6*, 25 °C): 3.06 (3H, s, C***H3***), 3.51 – 3.61 (4H, m, (C***H2)2***), 4.77 (1H, t, *J* = 5.30 Hz, O***H***), 6.84 (2H, d, *J* = 9.04 Hz, C***H*** (11&13)), 7.84 – 7.92 (6H, m, C***H*** (2&6, 3&5, 10&14), 8.01 (1H, s, C***H*** (8)). ^{13}C NMR: (100 MHz, DMSO-*d6*, 25 °C): δ = 38.74 (***C***H_3), 53.80 (HO-CH_2***C***H_2-N), 58.19 (HO-***C***H_2CH_2-N), 99.70 ***C***(7), 109.59 ***C***(1), 111.46 ***C***(11&13), 118.72 & 118.86 (2x***C***N), 120.03 ***C***(9), 125.40 ***C***(3&5), 131.94 ***C***(10&14), 132.89 ***C***(2&6), 139.65 ***C***(4), 145.41 ***C***(8), 151.59 ***C***(12).

[4-((3-Hydroxypropyl)(methyl)amino)-benzylidene]-4-cyanophenylacetonitrile (5)

Compound **5** was synthesized following general procedure B using compound **2** (610 mg, 3.20 mmol) and 4-cyanophenylacetonitrile (494 mg, 3.50 mmol) in 50 mL ethanol. Yield: 697 mg of compound **5** as an orange solid (70%). HR-ESI-MS (m/z): $[M+H]^+$ calcd.: 318.1606, found: 318.1587 ^1H NMR: (400 MHz, DMSO-*d6*, 25 °C): 1.70 (2H, p, *J* = 6.63 Hz, HO-CH_2C***H2***CH_2-N), 3.02 (3H, s, C***H3***), 3.44 – 3.52 (4H, m, HO-C***H2***CH_2C***H2***-N), 4.57 (1H, t, *J* = 4.98 Hz, O***H***), 6.82 (2H, d, *J* = 9.16 Hz, C***H*** (11&13)), 7.83 – 7.91 (6H, m, C***H*** (2&6, 3&5, 10&14), 7.99 (1H, s, C***H*** (8)). ^{13}C NMR: (100 MHz, DMSO-*d6*, 25 °C): δ = 29.61 (HO-CH_2***C***H_2CH_2-N), 37.98 (***C***H_3), 48.49 (HO-CH_2CH_2***C***H_2-N), 58.15 (HO-***C***$H_2CH_2CH_2$-N), 99.68 ***C***(7), 109.58 ***C***(1), 111.38 ***C***(11&13), 118.72 & 118.87 (2x***C***N), 119.99 ***C***(9), 125.39 ***C***(3&5), 132.01 ***C***(10&14), 132.87 ***C***(2&6), 139.66 ***C***(4), 145.40 ***C***(8), 151.35 ***C***(12).

[4-((2-(2-Hydroxyethoxy)ethyl)(methyl)amino)-benzylidene]-4-cyanophenylacetonitrile (6)

Compound **6** was synthesized following general procedure B using compound **3** (625 mg, 2.80 mmol) and 4-cyanophenylacetonitrile (438 mg, 3.10 mmol) in 45 mL ethanol. Yield: 447 mg of compound **6** as an orange solid (77%). HR-ESI-MS (m/z): $[M+H]^+$ calcd.: 348.1712, found: 348.1736. ^1H NMR: (400 MHz, DMSO-*d6*, 25 °C): δ = 3.05 (3H, s, ***H3***C), 3.43 (2H, m, (HO-CH_2C***H2***-O)), 3.48 (2H, m, (HO-C***H2***CH_2-O)), 3.61 (4H, m, (O-(C***H2)2***-N)), 4.57 (1H, t, *J* = 5.36 Hz, O***H***), 6.84 (2H, d, *J* = 9.16 Hz, C***H*** (11&13)), 7.84 – 7.92 (6H, m, C***H*** (2&6, 3&5, 10&14), 8.00 (1H, s, C***H*** (8)). ^{13}C NMR: (100 MHz, DMSO-*d6*, 25 °C): δ = 38.67 (***C***H_3), 51.07 (O-CH_2***C***H_2-N), 60.25 (HO-CH_2***C***H_2-N), 67.73 (O-***C***H_2CH_2-N), 72.39 (HO-CH_2***C***H_2-N), 99.95 ***C***(7), 109.66 ***C***(1), 111.56 ***C***(11&13), 118.72 & 118.82 (2x***C***N), 120.25 ***C***(9), 125.44 ***C***(3&5), 131.96 ***C***(10&14), 132.89 ***C***(2&6), 139.62 ***C***(4), 145.41 ***C***(8), 151.39 ***C***(12).

[4-((2-Bromoethyl)(methyl)amino)-benzylidene]-4-cyanophenylacetonitrile (7)

Compound **7** was synthesized following general procedure C using compound **4** (100 mg, 330 mmol), carbon tetrabromide (CBr_4, 164 mg, 495 µmol) and triphenylphosphine (PPh_3, 130 mg, 495 µmol) in 2.0 mL dichloromethane. Yield: 77.0 mg of compound **7** as an orange solid (64%). HR-ESI-MS (m/z): $[M+H]^+$ calcd.: 366.0606, found: 366.0554. ^{1}H NMR: (400 MHz, DMSO-d_6, 25 °C): 3.08 (3H, s, C$\boldsymbol{H_3}$), 3.65 (2H, t, J = 6.92, BrC$\boldsymbol{H_2}$CH$_2$NR$_2$), 3.87 (2H, t, J = 6.96, BrCH$_2$C$\boldsymbol{H_2}$NR$_2$), 6.88 (2H, d, J = 9.13 Hz, C***H*** (11&13)), 7.85 – 7.94 (6H, m, C***H*** (2&6, 3&5, 10&14), 8.03 (1H, s, C***H*** (8)). ^{13}C NMR: (100 MHz, DMSO-d_6, 25 °C): δ = 38.74 (***C***H$_3$), 53.80 (HO-CH$_2$***C***H$_2$-N), 58.19 (HO-***C***H$_2$CH$_2$-N), 99.70 ***C***(7), 109.59 ***C***(1), 111.46 ***C***(11&13), 118.72 & 118.86 (2x***C***N), 120.03 ***C***(9), 125.40 ***C***(3&5), 131.94 ***C***(10&14), 132.89 ***C***(2&6), 139.65 ***C***(4), 145.41 ***C***(8), 151.59 ***C***(12).

[4-((3-Bromopropyl)(methyl)amino)-benzylidene]-4-cyanophenylacetonitrile (8) [2]

Compound **8** was synthesized following general procedure C with compound **5** (105 mg, 331 µmol), carbon tetrabromide (CBr_4,165 mg, 496 µmol) and triphenylphosphine (PPh_3, 130 mg, 496 µmol) in 2.0 mL dichloromethane. Yield: 104 mg of compound **8** as an orange solid (83%). The characterization data of these compounds are in agreement with reported analysis given in ref. 2.

[4-((2-(2-Bromoethoxy)ethyl)(methyl)amino)-benzylidene]-4-cyanophenylacetonitrile (9)

Compound **9** was synthesized following HBC synthesis procedure C using compound **6** (127 mg, 365 µmol) carbon tetrabromide (CBr_4, 182 mg, 548 µmol) and triphenylphosphine (PPh_3, 144 mg, 548 µmol) in 2.0 mL dichloromethane Yield: 66.0 mg of compound **9** as an orange solid (44%). HR-ESI-MS (m/z): $[M+H]^+$ calcd.: 410.0868, found: 410.0899. ^{1}H NMR: (400 MHz, DMSO-d_6, 25 °C): δ = 3.07 (3H, s, $\boldsymbol{H_3}$C), 3.57 (2H, t, J = 5.61 Hz, (BrCH$_2$C$\boldsymbol{H_2}$O)), 3.65 (4H, s, (OC$\boldsymbol{H_2}$C$\boldsymbol{H_2}$-N)), 3.73 (2H, t, J = 5.63 Hz, BrC$\boldsymbol{H_2}$CH$_2$O)), 6.86 (2H, d, J = 9.11 Hz, C***H*** (11&13)), 7.84 – 7.92 (6H, m, C***H*** (2&6, 3&5, 10&14), 8.01 (1H, s, C***H*** (8)). ^{13}C NMR: (100 MHz, DMSO-d_6, 25 °C): δ = 32.34 (Br***C***H$_2$CH$_2$-O), 38.75 (***C***H$_3$), 51.00 (O-***C***H$_2$CH$_2$-N), 67.71 (O-CH$_2$***C***H$_2$-N), 70.31 (BrCH$_2$***C***H$_2$-O), 100.02 ***C***(7), 109.67 ***C***(1), 111.63 ***C***(11&13), 118.71 & 118.81 (2x***C***N), 120.31 ***C***(9), 125.46 ***C***(3&5), 131.95 ***C***(10&14), 132.90 ***C***(2&6), 139.61 ***C***(4), 145.42 ***C***(8), 151.36 ***C***(12).

(4-Hydroxybenzylidene)-4-cyanophenylacetonitrile (10)

4-Hydroxybenzaldehyde (122 mg, 1.00 mmol) was dissolved in 2.5 mL ethanol and mixed with piperidine (255 mg, 296 µL, 3.00 mmol) and stirred for 3 hours at room temperature whereby an orange solid precipitated. The orange solid was filtered off and washed two times with ice-cold ethanol. Yield: 220 mg of compound **10** as an orange solid (89%). HR-ESI-MS (m/z): $[M-H]^-$ calcd.: 245.0715, found: 245.0744. ^1H NMR: (400 MHz, DMSO-d_6, 25 °C): δ = 6.84 (2H, d, *J* = 8.78 Hz, C***H*** (11&13)), 7.86 - 7.93 (6H, m, C***H*** (2&6, 3&5, 10&14), 8.04 (1H, s, C***H*** (8)). ^{13}C NMR: (100 MHz, DMSO-d_6, 25 °C): δ = 102.08 ***C***(7), 110.13 ***C***(1), 116.63 ***C***(11&13), 118.39 & 118.63 (2x***C***N), 122.84 ***C***(9), 125.76 ***C***(3&5), 132.30 ***C***(10&14), 132.93 ***C***(2&6), 139.31 ***C***(4), 145.70 ***C***(8), 163.47 ***C***(12).

[4-(Bromobutoxy)-benzylidene]-4-cyanophenylacetonitrile (11)

Compound **10** (132 mg, 536 µmol) was dissolved in 850 µL dry tetrahydrofuran and cooled to 0 °C. Then, 4-bromo-1-butanol (123 mg, 73.0 µL, 804 µmol), triphenylphosphine (PPh_3, 211 mg, 804 µmol) and a 40% diethyl azodicarboxylate solution in toluene (DEAD, 350 mg, 366 µL, 804 µmol) were added and stirred for 30 minutes at 0 °C. After 30 minutes, the whole reaction mixture was loaded on a silica gel column and the product was eluted with 1% methanol in dichloromethane. Yield: 132 mg of compound **11** as a pale yellow solid (64%). ^1H NMR: (400 MHz, $CDCl_3$, 25 °C): δ = 1.97 – 2.13 (4H, m, $BrCH_2(C\boldsymbol{H_2})_2CH_2O$), 3.50 (2H, t, *J* = 6.48 Hz, $BrC\boldsymbol{H_2}(CH_2)_3O$), 4.08 (2H, t, *J* = 5.98 Hz, $Br(CH_2)_3C\boldsymbol{H_2}O$), 6.98 (2H, d, *J* = 8.90 Hz, C***H*** (11&13)), 7.55 (1H, s, C***H*** (8)). 7.74 (4H, q, *J* = 8.84 Hz, C***H*** (2&6, 3&5), 7.92 (2H, d, *J* = 8.78 Hz, C***H*** (10&14)). ^{13}C NMR: (100 MHz, $CDCl_3$, 25 °C): δ = 27.86 ($BrCH_2\boldsymbol{C}H_2(CH_2)_2O$), 29.45 ($Br(CH_2)_2\boldsymbol{C}H_2CH_2O$), 33.38 $Br\boldsymbol{C}H_2(CH_2)_3O$, 67.32 ($Br(CH_2)_3\boldsymbol{C}H_2O$), 106.74 ***C***(7), 112.21 ***C***(1), 115.17 ***C***(11&13), 117.88 & 118.47 (2x***C***N), 125.91 ***C***(9), 126.34 ***C***(3&5), 131.95 ***C***(10&14), 132.90 ***C***(2&6), 139.37 ***C***(4), 144.51 ***C***(8), 161.67 ***C***(12).

[4-((3-Chloropropyl)(methyl)amino)-benzylidene]-4-cyanophenylacetonitrile (12)

Compound **5** (57.0 mg, 180 µmol), *N*-chlorosuccinimide (NCS, 31.2 mg, 234 µmol) and triphenylphosphine (PPh_3, 61.2 mg, 234 µmol) were dissolved in 1.8 mL tetrahydrofuran and stirred for 48 hours at room temperature. Then, the whole mixture was loaded on a small silica column and the pure compound was eluted using 100% dichloromethane. Yield: 52.0 mg of compound **12** as an orange solid (86%). HR-ESI-MS (m/z): $[M+H]^+$ calcd.: 336.1268, found: 336.1266 ^1H NMR: (400 MHz, $CDCl_3$, 25 °C): 2.10 (2H, m, $ClCH_2C\boldsymbol{H_2}CH_2N$), 3.09 (3H, s, $C\boldsymbol{H_3}$), 3.61 (4H, m, $ClC\boldsymbol{H_2}CH_2C\boldsymbol{H_2}N$), 6.75 (2H, d, *J* = 9.08 Hz, C***H*** (11&13)), 7.48 (1H, s, C***H***(8)). 7.69 (4H, m, C***H*** (2&6, 3&5), 7.88 (2H, d, *J* = 9.01 Hz, C***H*** (10&14)). ^{13}C NMR: (100 MHz, DMSO-

d$_6$, 25 °C): δ = 29.94 ($ClCH_2\mathbf{C}H_2CH_2N$), 38.84 (**C**H_3), 42.49 ($Cl\mathbf{C}H_2CH_2CH_2N$), 49.49 ($ClCH_2CH_2\mathbf{C}H_2N$), 102.44 ***C***(7), 111.15 ***C***(1), 111.71 ***C***(11&13), 118.76 & 118.81 (2x**C**N), 121.14 ***C***(9), 125.83 ***C***(3&5), 132.31 ***C***(10&14), 132.77 ***C***(2&6), 140.18 ***C***(4), 144.76 ***C***(8), 151.30 ***C***(12).

[4-((3-Iodopropyl)(methyl)amino)-benzylidene]-4-cyanophenylacetonitrile (13)

Compound **5** (56.0 mg, 176 µmol) was dissolved in 1.8 mL tetrahydrofuran and cooled to 0 °C. Then, iodine (I_2, 58.2 mg, 229 µmol), imidazole (19.2 mg, 282 µmol) and triphenylphosphine (PPh_3, 62.5 mg, 238 µmol) were added and stirred under argon atmosphere for 48 hours at room temperature. The resulting mixture was loaded on a small silica column and the pure compound was eluted using pure dichloromethane. Yield: 73.0 mg of compound **13** as an orange solid (97%). HR-ESI-MS (m/z): 428.0624, found: 428.0601. ^{1}H NMR: (400 MHz, $CDCl_3$, 25 °C): 2.14 (2H, p, *J* = 6.82 Hz, $ICH_2C\mathbf{H_2}CH_2N$), 3.10 (3H, s, C$\mathbf{H_3}$), 3.21 (2H, t, *J* = 6.57 Hz, $IC\mathbf{H_2}CH_2CH_2N$), 3.56 (2H, t, *J* = 7.08 Hz, $ICH_2CH_2C\mathbf{H_2}N$), 6.75 (2H, d, *J* = 9.09 Hz, C***H*** (11&13)), 7.48 (1H, s, C***H***(8)). 7.70 (4H, q, *J* = 9.21 Hz, C***H*** (2&6, 3&5), 7.88 (2H, d, *J* = 8.99 Hz, C***H*** (10&14)). ^{13}C NMR: (100 MHz, $CDCl_3$, 25 °C): δ = 2.84 ($I\mathbf{C}H_2CH_2CH_2N$), 30.71 ($ICH_2\mathbf{C}H_2CH_2N$), 38.96 (**C**H_3), 52.70 ($ICH_2CH_2\mathbf{C}H_2N$), 102.51 ***C***(7), 111.19 ***C***(1), 111.78 ***C***(11&13), 118.77 & 118.81 (2x**C**N), 121.20 ***C***(9), 125.86 ***C***(3&5), 132.31 ***C***(10&14), 132.79 ***C***(2&6), 140.19 ***C***(4), 144.76 ***C***(8), 151.29 ***C***(12).

[4-((3-(Mesyloxy)propyl)(methyl)amino)-benzylidene]-4-cyanophenylacetonitrile (14) [2]

Compound **14** was synthesized following general procedure D using compound **5** (51.0 mg, 161 µmol), triethylamine (NEt_3, 48.8 mg, 67.2 µL, 482 µmol) and methanesulfonylchloride (MsCl, 27.6 mg, 18.7 µL, 241 µmol) in 4.0 mL dichloromethane. Yield: 56.0 mg of compound **14** as an orange solid (88%). The characterization data of these compounds are in agreement with reported analysis given in ref. 2.

[4-((3-(Tosyloxy)propyl)(methyl)amino)-benzylidene]-4-cyanophenylacetonitrile (15)

To a solution containing compound **5** (95.0 mg, 299 µmol), triethylamine (NEt_3, 38.3 mg, 52.7 µL, 378 µmol) and 4-(dimethylamino)pyridine (DMAP, 1.80 mg, 15.0 µmol) in 1.0 mL dichloromethane, was added 4-toluenesulfonyl chloride (TsCl, 59.9 mg, 315 µmol) and the mixture was stirred at room temperature for 16 hours. This mixture was poured onto a silica gel column, and the pure compound was eluted with pure dichloromethane. Yield: 22.0 mg of compound **15** as an orange solid (16%). HR-ESI-MS (m/z): $[M+H]^+$ calcd.: 472.1694, found:

472.1741. ^{1}H NMR: (400 MHz, $CDCl_3$, 25 °C): 1.97 (2H, p, *J* = 6.37, $TsOCH_2C\boldsymbol{H_2}CH_2NR_2$), 2.45 (3H, s, $C\boldsymbol{H_3}$ (Ts)), 3.01 (3H, s, N-$C\boldsymbol{H_3}$), 3.52 (2H, t, *J* = 6.99, $TsOCH_2CH_2C\boldsymbol{H_2}NR_2$), 4.10 (2H, t, *J* = 5.79, $TsOC\boldsymbol{H_2}CH_2CH_2NR_2$), 6.66 (2H, d, *J* = 9.08 Hz, C***H*** (11&13)), 7.36 (2H, d, *J* = 8.11 Hz, C***H***(Ts)), 7.47 (1H, s, C***H*** (8)). 7.69 (4H, q, *J* = 9.35 Hz, C***H*** (2&6, 3&5), 7.79 (2H, d, *J* = 8.32 Hz, C***H***(Ts)), 7.85 (2H, d, *J* = 9.04 Hz, C***H*** (10&14)). ^{13}C NMR: (100 MHz, $CDCl_3$, 25 °C): δ = 21.80 ($TsOCH_2\boldsymbol{C}H_2CH_2NR_2$), 26.71 (C-***C***$H_3$), 38.79 (N-***C***$H_3$), 48.59 ($TsOCH_2CH_2\boldsymbol{C}H_2NR_2$), 67.85 ($TsO\boldsymbol{C}H_2CH_2CH_2NR_2$), 102.53 ***C***(7), 111.18 ***C***(1), 111.69 ***C***(11&13), 118.74 & 118.77 (2x***C***N), 121.19 ***C***(9), 125.83 ***C***(3&5), 128.02 & 130.10 ***C***H (Ts), 132.27 ***C***(10&14), 132.77 ***C***(2&6), 132.89 ***C***-CH_3, 140.12 ***C***(4), 144.72 ***C***(8), 145.21 ***C***-S(Ts), 151.09 ***C***(12).

[4-((2-(Mesyloxy)ethyl)(methyl)amino)-benzylidene]-4-cyanophenylacetonitrile (16)

Compound **16** was synthesized following general procedure D using compound **4** (42.2 mg, 139 µmol), triethylamine (NEt_3, 42.2 mg, 58.2 µL, 417 µmol) and methanesulfonylchloride (MsCl, 23.9 mg, 16.2 µL, 209 µmol) in 4.0 mL dichloromethane. Yield: 48.0 mg of compound **16** as an orange solid (90%). HR-ESI-MS (m/z): $[M+H]^+$ calcd.: 382.1225, found: 382.1200. ^{1}H NMR: (400 MHz, $CDCl_3$, 25 °C): δ = 2.97 (3H, s, S-$C\boldsymbol{H_3}$), 3.13 (3H, s, N-$C\boldsymbol{H_3}$), 3.82 (2H, t, *J* = 5.78, $MsOCH_2C\boldsymbol{H_2}NR_2$), 4.41 (2H, t, *J* = 5.77, $MsOC\boldsymbol{H_2}CH_2NR_2$), 6.76 (2H, d, *J* = 9.08 Hz, C***H*** (11&13)), 7.49 (1H, s, C***H*** (8)). 7.70 (4H, q, *J* = 9.02 Hz, C***H*** (2&6, 3&5), 7.89 (2H, d, *J* = 8.98 Hz, C***H*** (10&14)). ^{13}C NMR: (100 MHz, $CDCl_3$, 25 °C): δ = 37.75 (S-***C***H_3), 39.22 (N-***C***H_3), 51.27 ($MsOCH_2\boldsymbol{C}H_2NR_2$), 66.12 ($MsO\boldsymbol{C}H_2CH_2NR_2$), 103.41 ***C***(7), 111.40 ***C***(1), 111.92 ***C***(11&13), 118.60 & 118.70 (2x***C***N), 121.93 ***C***(9), 125.94 ***C***(3&5), 132.27 ***C***(10&14), 132.81 ***C***(2&6), 139.95 ***C***(4), 144.60 ***C***(8), 150.87 ***C***(12).

[4-((2-((2-Mesyloxy)ethoxy)ethyl)(methyl)amino)-benzylidene]-4-cyanophenylacetonitrile (17)

Compound **17** was synthesized following general procedure D using compound **6** (50.1 mg, 150 µmol), triethylamine (NEt_3, 45.5 mg, 62.0 µL, 450 µmol) and methanesulfonylchloride (MsCl, 25.7 mg, 17.4 µL, 224.8 µmol) in 4.0 mL dichloromethane. Yield: 56.0 mg of compound **17** as an orange solid (98%). HR-ESI-MS (m/z): $[M+H]^+$ calcd.: 426.1488, found: 426.1523. ^{1}H NMR: (400 MHz, $CDCl_3$, 25 °C): δ = 2.99 (3H, s, $\boldsymbol{H_3}$C-S), 3.11 (3H, s, $\boldsymbol{H_3}$C-N), 3.64 - 3.74 (6H, m, ($MsOCH_2C\boldsymbol{H_2}O$ & $OC\boldsymbol{H_2}C\boldsymbol{H_2}$-N)), 4.34 (2H, m, $MsOC\boldsymbol{H_2}CH_2O$)), 6.74 (2H, d, *J* = 9.09 Hz, C***H*** (11&13)), 7.48 (1H, s, C***H***(8)). 7.70 (4H, q, *J* = 9.06 Hz, C***H*** (2&6, 3&5), 7.87 (2H, d, *J* = 9.00 Hz, C***H*** (10&14)). ^{13}C NMR: (100 MHz, $CDCl_3$, 25 °C): δ = 37.70 (S-***C***H_3), 39.24 (N-***C***H_3), 52.04 (O-$CH_2\boldsymbol{C}H_2$-N), 68.88, 68.95 & 69.21 ($MsO\boldsymbol{C}H_2\boldsymbol{C}H_2$-O & O-$\boldsymbol{C}H_2CH_2$-N), 100.46

C(7), 111.17 ***C***(1), 111.78 ***C***(11&13), 118.76 & 118.82 (2x***C***N), 121.16 ***C***(9), 125.84 ***C***(3&5), 132.24 ***C***(10&14), 132.79 ***C***(2&6), 140.17 ***C***(4), 144.77 ***C***(8), 151.48 ***C***(12).

[4-((4-Mesyloxy)butoxy)-benzylidene]-4-cyanophenylacetonitrile (18)

Compound **11** (100 mg, 262 µmol) was suspended in 1.5 mL DMSO/DMF/H_2O 1:1:1 and 300 µL conc. hydrochloric acid were added and the mixture stirred at 100 °C for 72 hours. The resulting slightly yellow colored solution was evaporated under high vacuum to remove DMF and water. The remaining dimethyl sulfoxide was dissolved in dichloromethane and extracted first with ice-cold water, second with saturated bicarbonate solution and last with brine. To remove last traces of dimethyl sulfoxide, a silica gel column was prepared and the crude intermediate was washed with pure dichloromethane and finally eluted with 2% methanol in dichloromethane. Further functionalization was achieved following general procedure D using the previously prepared intermediate (63.0 mg, ≈ 198 µmol), triethylamine (NEt_3, 60.1 mg, 82.7 µL, 594 µmol) and methanesulfonylchloride (MsCl, 34.0 mg, 23.0 µL, 296.8 µmol) in 4.0 mL dichloromethane. Yield: 32.0 mg of compound **18** as a pale yellow solid (41%). ^{1}H NMR: (400 MHz, $CDCl_3$, 25 °C): δ = 1.98 (4H, m, $MsOCH_2(C\boldsymbol{H_2})_2CH_2O$), 3.03 (3H, s, S-C***H₃***), 4.09 (2H, t, *J* = 5.55 Hz, $MsOCH_2(CH_2)_2C\boldsymbol{H_2}O$), 4.33 (2H, t, *J* = 5.88 Hz, $MsOC\boldsymbol{H_2}(CH_2)_2CH_2O$), 6.98 (2H, d, *J* = 8.88 Hz, C***H*** (11&13)), 7.55 (1H, s, C***H*** (8)). 7.74 (4H, q, *J* = 8.70 Hz, C***H*** (2&6, 3&5), 7.91 (2H, d, *J* = 8.85 Hz, C***H*** (10&14)). ^{13}C NMR: (100 MHz, $CDCl_3$, 25 °C): δ = 25.41 ($MsO(CH_2)_2\boldsymbol{C}H_2CH_2O$), 26.19 ($MsOCH_2\boldsymbol{C}H_2(CH_2)_2O$), 67.37 ($MsO(CH_2)_3\boldsymbol{C}H_2O$), 69.52 ($MsO\boldsymbol{C}H_2(CH_2)_3O$), 106.87 ***C***(7), 112.26 ***C***(1), 115.17 ***C***(11&13), 117.87 & 118.48 (2x***C***N), 126.01 ***C***(9), 126.36 ***C***(3&5), 131.97 ***C***(10&14), 132.92 ***C***(2&6), 139.36 ***C***(4), 144.47 ***C***(8), 161.57 ***C***(12).

[4-((3-(*tert*-Butyldimethylsilyloxy)propyl)(methyl)amino)-benzylidene]-4-iodophenylacetonitrile (19)

Step i: Compound **2** (449 mg, 2.30 mmol), 4-iodophenylacetonitrile (621 mg, 2.60 mmol) and piperidine (593 mg, 689 µL) were dissolved in 5.2 mL ethanol and stirred at 80 °C for 16 hours. After evaporation of the solvent, the crude intermediate was purified using silica gel chromatography 50–60% ethyl acetate in toluene. Step ii: The remaining oil (811 mg) was dissolved in 7.0 mL dichloromethane, then *tert*-butyldimethylsilyl chloride (TBSCl, 585 mg, 3.90 mmol) and imidazole (528 mg, 7.80 mmol) were added whereby a white precipitate was formed. This suspension was stirred for two hours at room temperature followed by washing with half saturated bicarbonate solution and brine. Lastly, the organic layer was dried over Na_2SO_4, filtrated and concentrated to a volume of 2.0 mL and purified by silica gel

chromatography using 100% dichloromethane. Yield: 947 mg of compound **19** as a yellow solid (77%). HR-ESI-MS (m/z): $[M+H]^+$ calcd.: 533.1485, found: 533.1449. ^{1}H NMR: (400 MHz, $CDCl_3$, 25 °C): 0.08 (6H, s, $Si(CH_3)_2$), 0.94 (9H, s, $SiC(C\boldsymbol{H_3})_3$), 1.81 (2H, p, J = 6.40 Hz, $TBSOCH_2C\boldsymbol{H_2}CH_2$-N), 3.04 (3H, s, $C\boldsymbol{H_3}$), 3.53 (2H, t, J = 7.12 Hz, $TBSOCH_2CH_2C\boldsymbol{H_2}$-N), 3.67 (2H, t, J = 5.70 Hz, $TBSOC\boldsymbol{H_2}CH_2CH_2$-N), 6.73 (2H, d, J = 9.05 Hz, C***H*** (11&13)), 7.37 (3H, m, C***H*** (8 & 3&5), 7.71 (2H, d, J = 8.62 Hz, C***H*** (2&6)), 7.83 (2H, d, J = 9.00 Hz, C***H*** (10&14)). ^{13}C NMR: (100 MHz, $CDCl_3$, 25 °C): δ = -5.22 ($Si(\boldsymbol{C}H_3)_2$), 18.40 ($Si\boldsymbol{C}(CH_3)_2$), 26.05 ($SiC(\boldsymbol{C}H_3)_2$), 30.14 ($TBSOCH_2\boldsymbol{C}H_2CH_2N$), 38.55 ($\boldsymbol{C}H_3$), 49.14 ($TBSOCH_2CH_2\boldsymbol{C}H_2N$), 60.24 ($TBSO\boldsymbol{C}H_2CH_2CH_2$-N), 93.25 ***C***(1), 102.98 ***C***(7), 111.59 ***C***(11&13), 119.26 (***C***N), 121.07 ***C***(9), 127.19 ***C***(3&5), 131.72 ***C***(10&14), 135.47 ***C***(2&6), 138.03 ***C***(4), 142.92 ***C***(8), 151.14 ***C***(12).

[4-((3-(*tert*-Butyldimethylsilyloxy)propyl)(methyl)amino)-benzylidene]-4-vinylphenyl-acetonitrile (20)

Compound **19** (406 mg, 762 µmol) was dissolved in 15 mL dry and degassed *N,N*-dimethylformamide under argon atmosphere. Then, vinyltributyltin (266 mg, 245 µL, 839 µmol) and the catalyst bis(triphenylphosphine)palladium(II) dichloride ($Pd(PPh_3)_2Cl_2$, 26.8 mg, 38.1 µmol) were added and the whole was stirred for two hours at 60 °C. After two hours, reaction control by thin layer chromatography showed complete turnover of the starting material. Water was added to the reaction mixture and the resulting solution was transferred into a separatory funnel, extracted three times with dichloromethane, dried over Na_2SO_4 and concentrated to dryness. The crude compound was purified via silica gel chromatography using 50–100% dichloromethane in cyclohexane. Yield: 319 mg of compound **20** as a yellow oil (97%). TLC: cyclohexane/dichloromethane 6/4 R_f = 0.58. HR-ESI-MS (m/z): $[M+H]^+$ calcd.: 433.2675, found: 433.2722. ^{1}H NMR: (400 MHz, $CDCl_3$, 25 °C): 0.08 (6H, s, $Si(CH_3)_2$), 0.94 (9H, s, $SiC(C\boldsymbol{H_3})_3$), 1.81 (2H, p, J = 6.42 Hz, $TBSOCH_2C\boldsymbol{H_2}CH_2N$), 3.04 (3H, s, $C\boldsymbol{H_3}$), 3.53 (2H, t, J = 7.13 Hz, $TBSOCH_2CH_2C\boldsymbol{H_2}N$), 3.67 (2H, t, J = 5.73 Hz, $TBSOC\boldsymbol{H_2}CH_2CH_2$-N), 5.28 & 5.78 (2H, dxd, J = 36.01 Hz, $CH{=}C\boldsymbol{H_2}$ (vinyl)), 6.73 (3H, m, C***H*** (11&13) & C***H***$=CH_2$ (vinyl)), 7.40 (1H, s, C***H*** (8), 7.43 (2H, d, J = 8.37 Hz, C***H*** (3&5)), 7.59 (2H, d, J = 8.45 Hz, C***H*** (2&6)), 7.84 (2H, d, J = 8.96 Hz, C***H*** (10&14)). ^{13}C NMR: (100 MHz, $CDCl_3$, 25 °C): δ = -5.22 ($Si(\boldsymbol{C}H_3)_2$), 18.40 ($Si\boldsymbol{C}(CH_3)_2$), 26.05 ($SiC(\boldsymbol{C}H_3)_2$), 30.14 ($TBSOCH_2\boldsymbol{C}H_2CH_2N$), 38.55 ($\boldsymbol{C}H_3$), 49.15 ($TBSOCH_2CH_2\boldsymbol{C}H_2N$), 60.27 ($TBSO\boldsymbol{C}H_2CH_2CH_2N$), 103.94 ***C***(7), 111.59 ***C***(11&13), 114.47 ($CH{=}\boldsymbol{C}H_2$ (vinyl)), 119.57 (***C***N), 121.40 ***C***(9), 125.61 ***C***(2&6), 126.81 ***C***(3&5), 131.57 ***C***(10&14), 135.16 ***C***(4), 136.25 ($\boldsymbol{C}H{=}CH_2$ (vinyl)), 137.30 ***C***(1), 142.21 ***C***(8), 150.93 ***C***(12).

[4-((3-Hydroxypropyl)(methyl)amino)-benzylidene]-styrenyl-acetonitrile (21)

Compound **20** (319 mg, 737 µmol) and tetrabutylammonium fluoride trihydrate (TBAF·3H_2O, 465 mg, 1.50 mmol) were dissolved in 3.8 mL tetrahydrofuran and stirred at room temperature for one hour. Then, the solvent was removed and the crude compound was purified using silica gel chromatography with 0–2% methanol in dichloromethane. Yield: 223 mg of compound **21** as an orange solid (95%). HR-ESI-MS (m/z): $[M+H]^+$ calcd.: 319.18, found: 319.18. ^{1}H NMR: (400 MHz, DMSO-d_6, 25 °C): 1.69 (2H, p, J = 6.64 Hz, $HOCH_2C\boldsymbol{H_2}CH_2N$), 3.00 (3H, s, $C\boldsymbol{H_3}$), 3.47 (4H, p, J = 6.26 Hz, $TBSOC\boldsymbol{H_2}CH_2C\boldsymbol{H_2}N$), 4.56 (1H, t, J = 4.99 Hz, ***H***O), 5.28 & 5.89 (2H, dxd, J = 49.98 Hz, $CH{=}C\boldsymbol{H_2}$ (vinyl)), 6.76 (3H, m, C***H*** (11&13) & C***H***=CH_2 (vinyl)), 7.55 (2H, d, J = 8.47 Hz, C***H*** (3&5)), 7.66 (2H, d, J = 8.47 Hz, C***H*** (2&6)), 7.81 (1H, s, C***H*** (8), 7.86 (2H, d, J = 9.06 Hz, C***H*** (10&14)). ^{13}C NMR: (100 MHz, DMSO-d_6, 25 °C): δ = 29.57 ($HOCH_2\boldsymbol{C}H_2CH_2N$), 37.93 ($\boldsymbol{C}H_3$), 48.47 ($HOCH_2CH_2\boldsymbol{C}H_2N$), 58.20 ($HO\boldsymbol{C}H_2CH_2CH_2N$), 101.60 ***C***(7), 111.33 ***C***(11&13), 114.83 ($CH{=}\boldsymbol{C}H_2$ (vinyl)), 119.29 (***C***N), 120.48 ***C***(9), 125.06 ***C***(2&6), 126.76 ***C***(3&5), 131.30 ***C***(10&14), 134.34 ***C***(4), 135.90 ($\boldsymbol{C}H{=}CH_2$ (vinyl)), 136.60 ***C***(1), 142.51 ***C***(8), 150.73 ***C***(12).

[4-((3-Mesyloxypropyl)(methyl)amino)-benzylidene]-(4-vinylphenyl)acetonitrile (22) [2]

Compound **22** was synthesized following general procedure D using compound **21** (274 mg, 860 µmol), triethylamine (NEt_3, 261 mg, 360 µL, 2.60 mmol) and methanesulfonylchloride (MsCl, 148 mg, 100 µL, 1.30 mmol) in 20 mL dichloromethane. Yield: 316 mg of compound **11** as an orange solid (93%). The characterization data of these compounds are in agreement with reported analysis given in ref. 2.

References

1. Chen, X.; Zhang, D.; Su, N.; Bao, B.; Xie, X.; Zuo, F.; Yang, L.; Wang, H.; Jiang, L.; Lin, Q.; Fang, M.; Li, N.; Hua, X.; Chen, Z.; Bao, C.; Xu, J.; Du, W.; Zhang, L.; Zhao, Y.; Zhu, L.; Loscalzo, J.; Yang, Y. *Nat. Biotechnol.* **2019,** *37,* 1287–1293. doi:10.1038/s41587-019-0249-1
2. Bereiter, R.; Flemmich, L.; Nykiel, K.; Heel, S.; Geley, S.; Hanisch, M; Eichler, C.; Breuker, K.; Lusser, A.; Micura, R. *Nat. Chem. Biol.* **2025,** doi:10.1038/s41589-024-01801-3

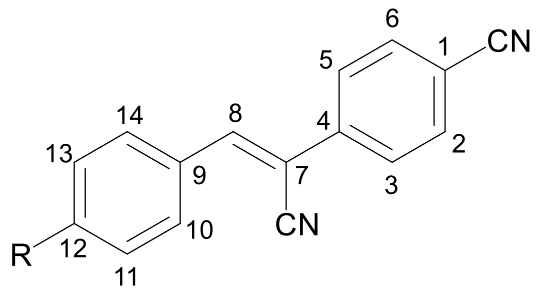

Carbon numbering used for peak assignments in the ^{1}H and ^{13}C NMR spectra when a HBC core is present.

NMR spectra of compounds

^{1}H NMR (400 MHz, DMSO-d_6, 25 °C) of compound **1**

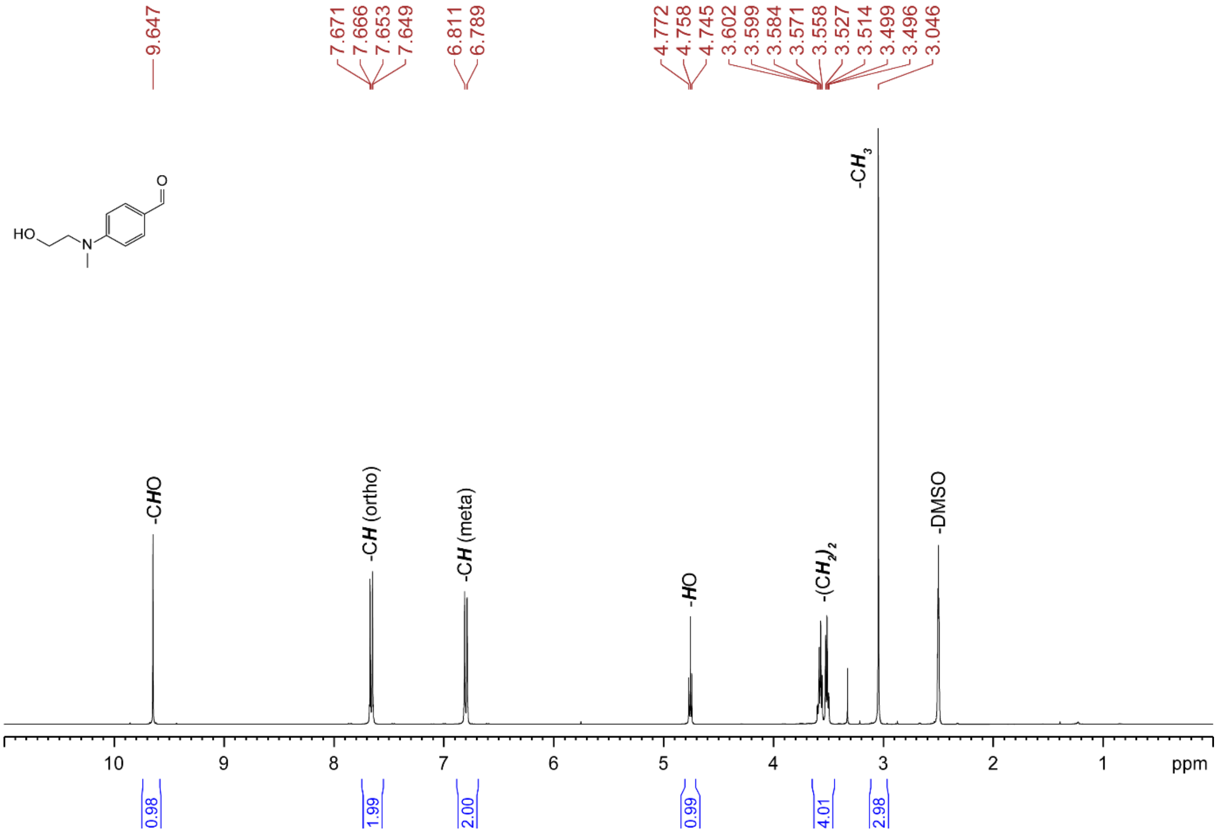

^{13}C NMR (100 MHz, DMSO-d_6, 25 °C) of compound **1**

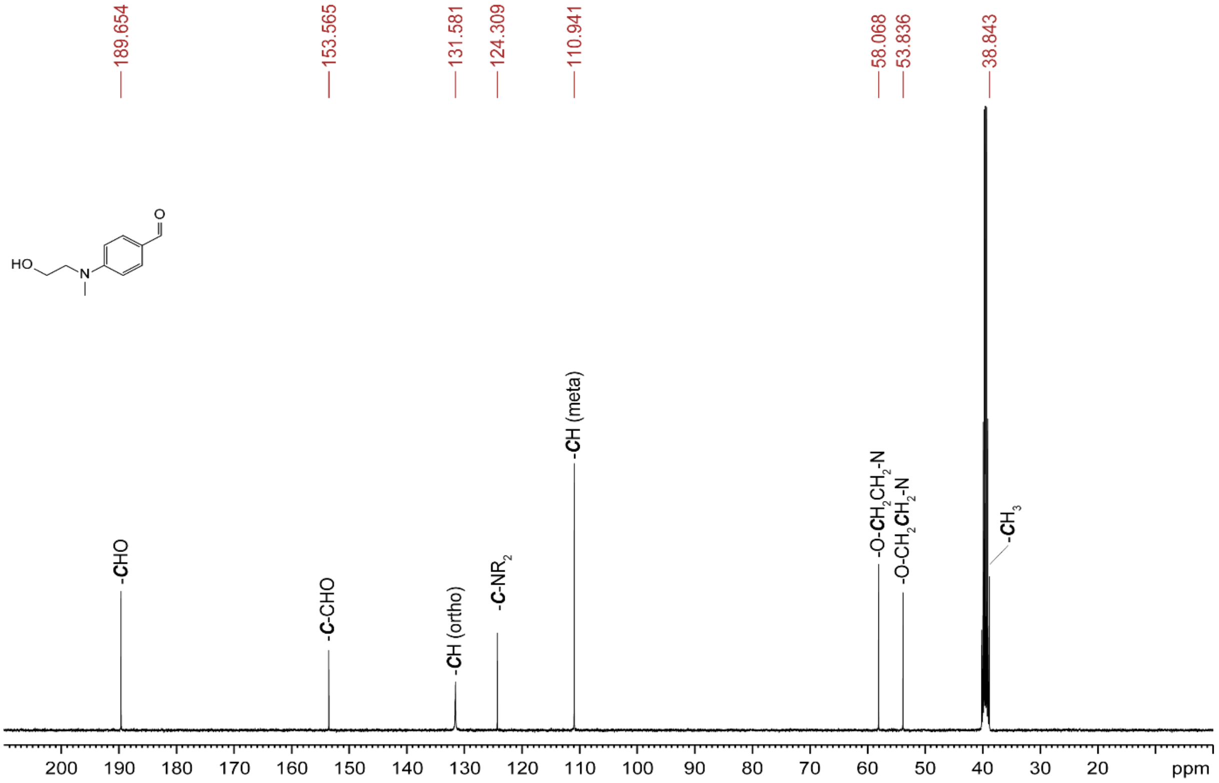

^1H NMR (400 MHz, DMSO-d_6, 25 °C) of compound **2**

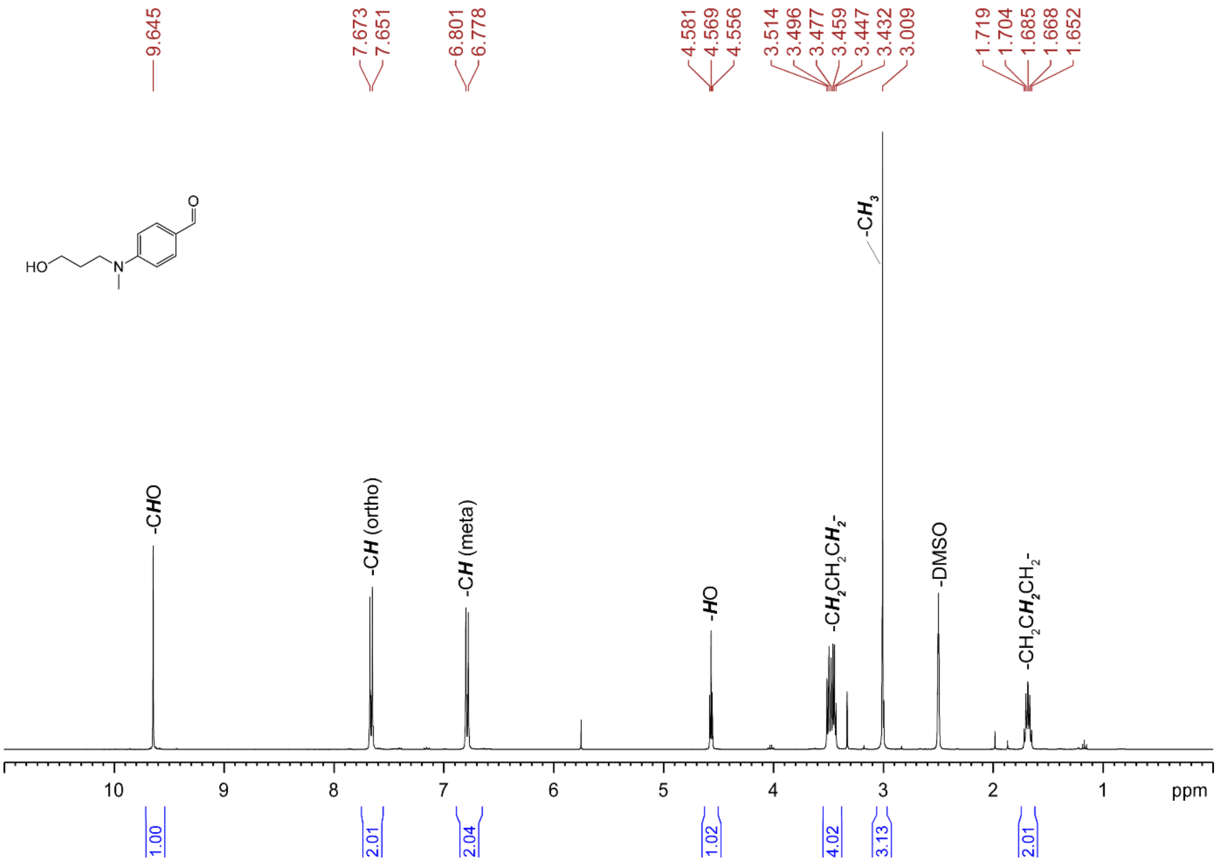

^{13}C NMR (100 MHz, DMSO-d_6, 25 °C) of compound **2**

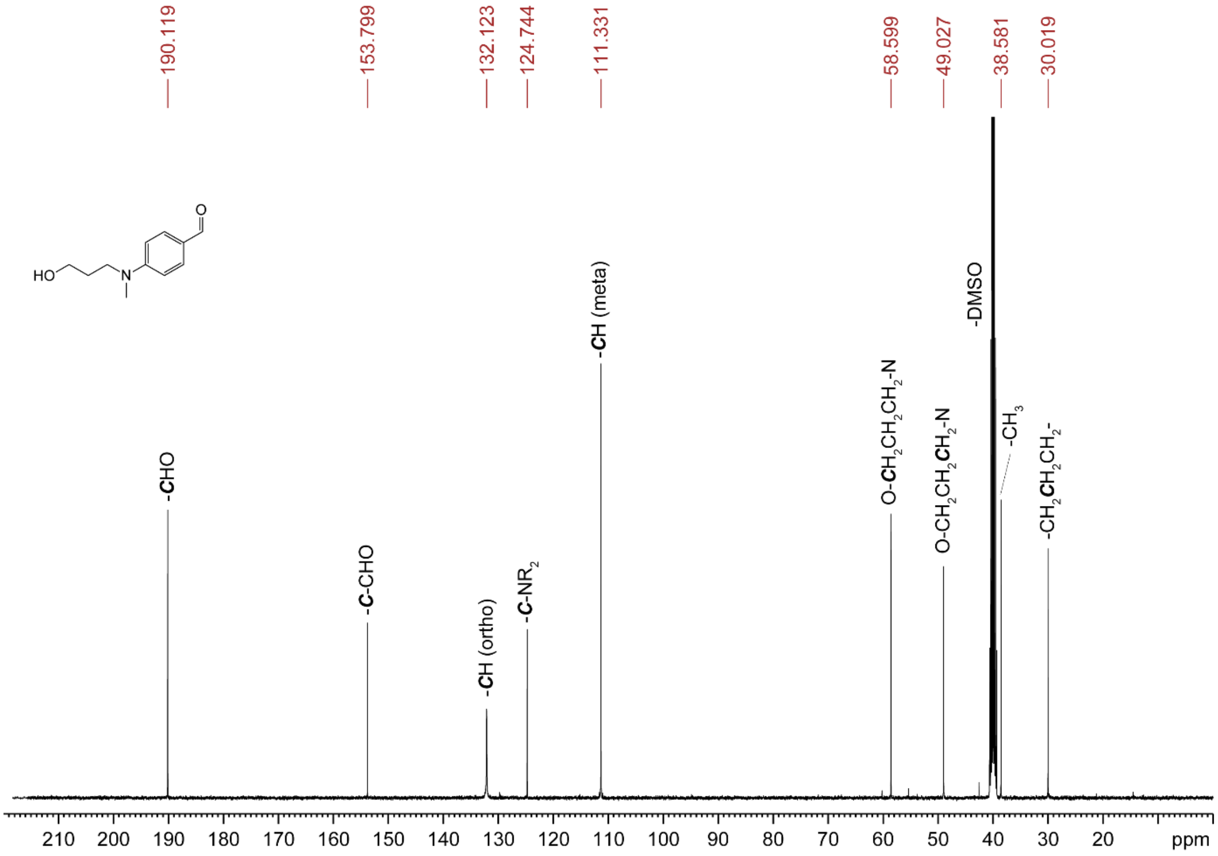

^{1}H NMR (400 MHz, DMSO-d_6, 25 °C) of compound **3**

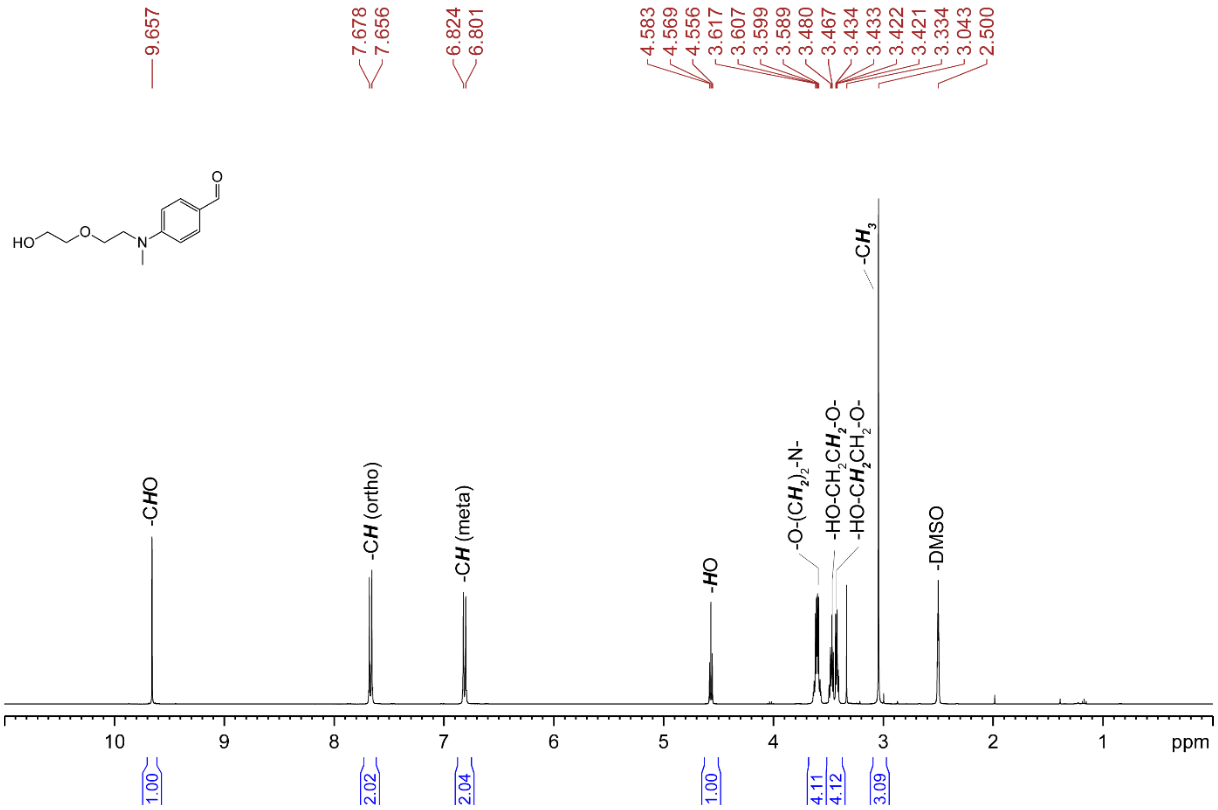

^{13}C NMR (100 MHz, DMSO-d_6, 25 °C) of compound **3**

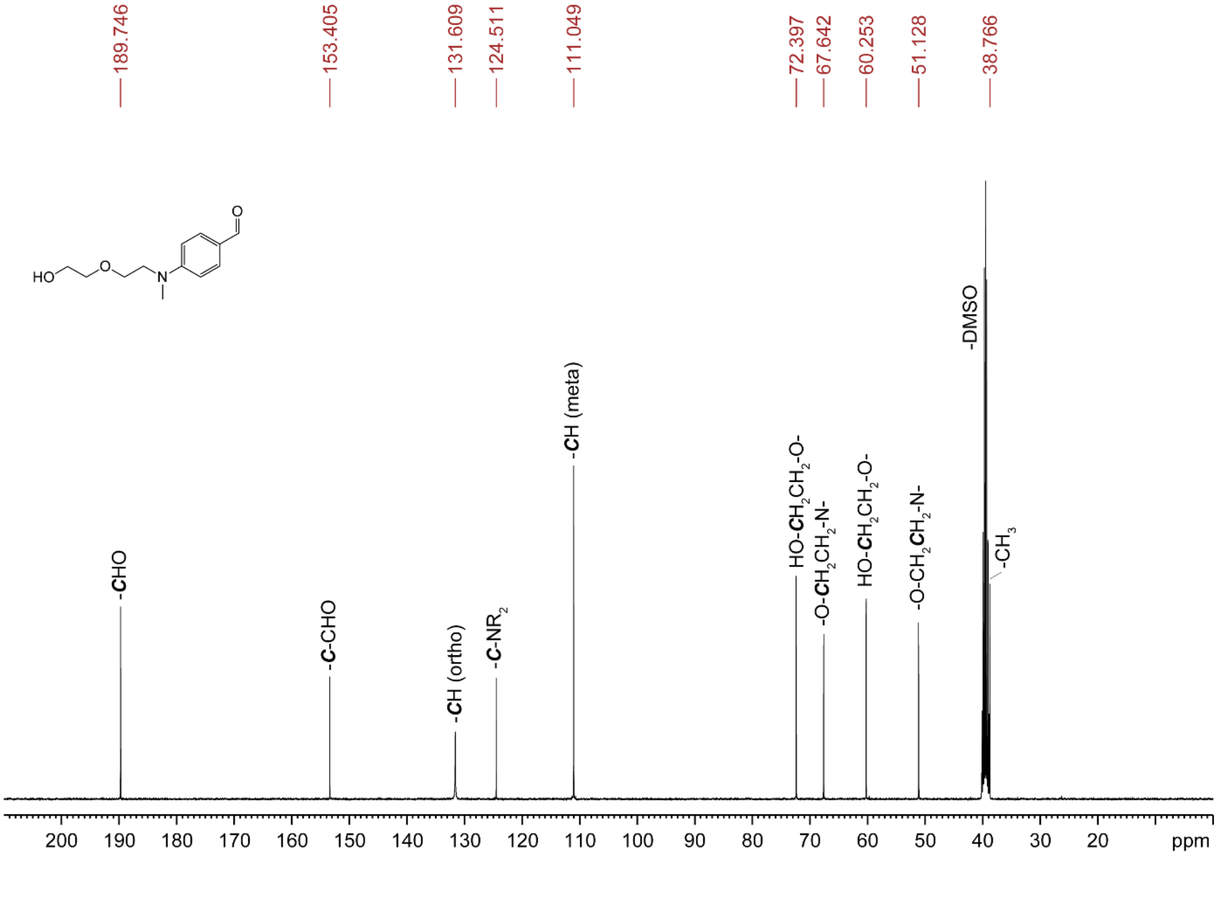

^{1}H NMR (400 MHz, DMSO-d_6, 25 °C) of compound **4**

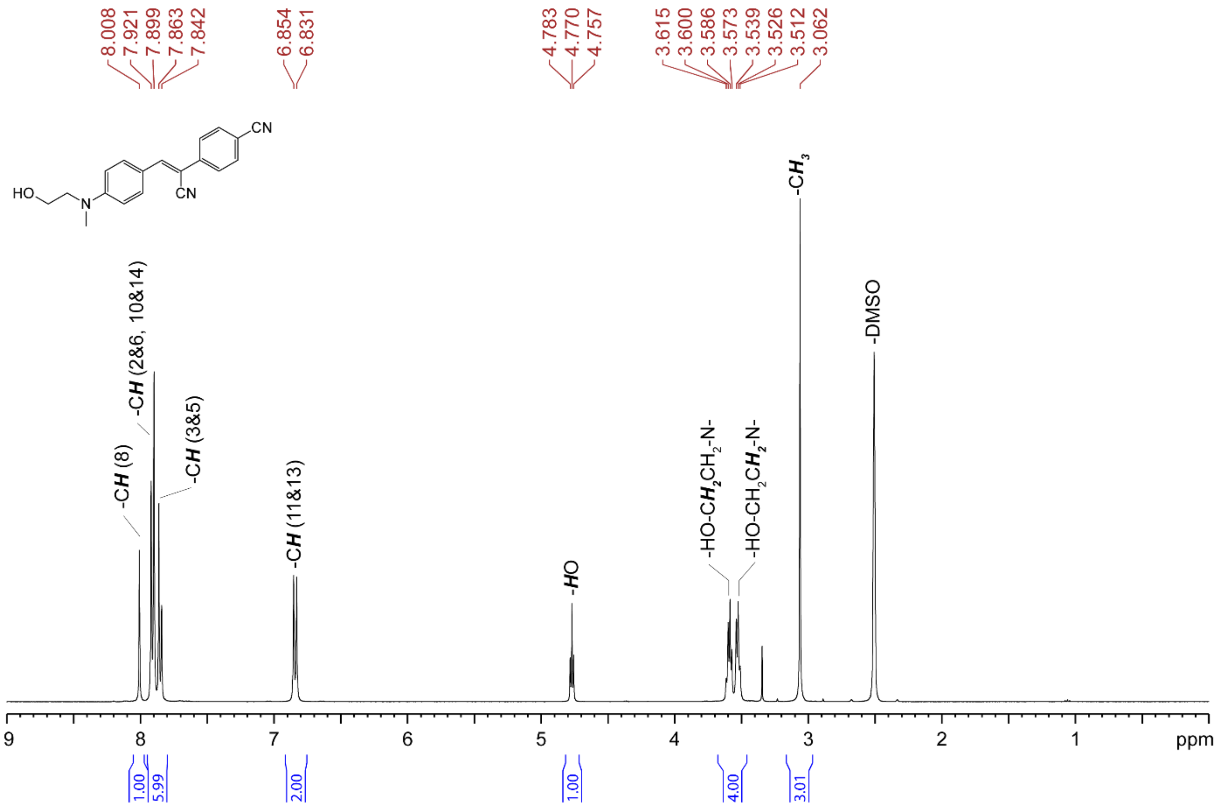

^{13}C NMR (100 MHz, DMSO-d_6, 25 °C) of compound **4**

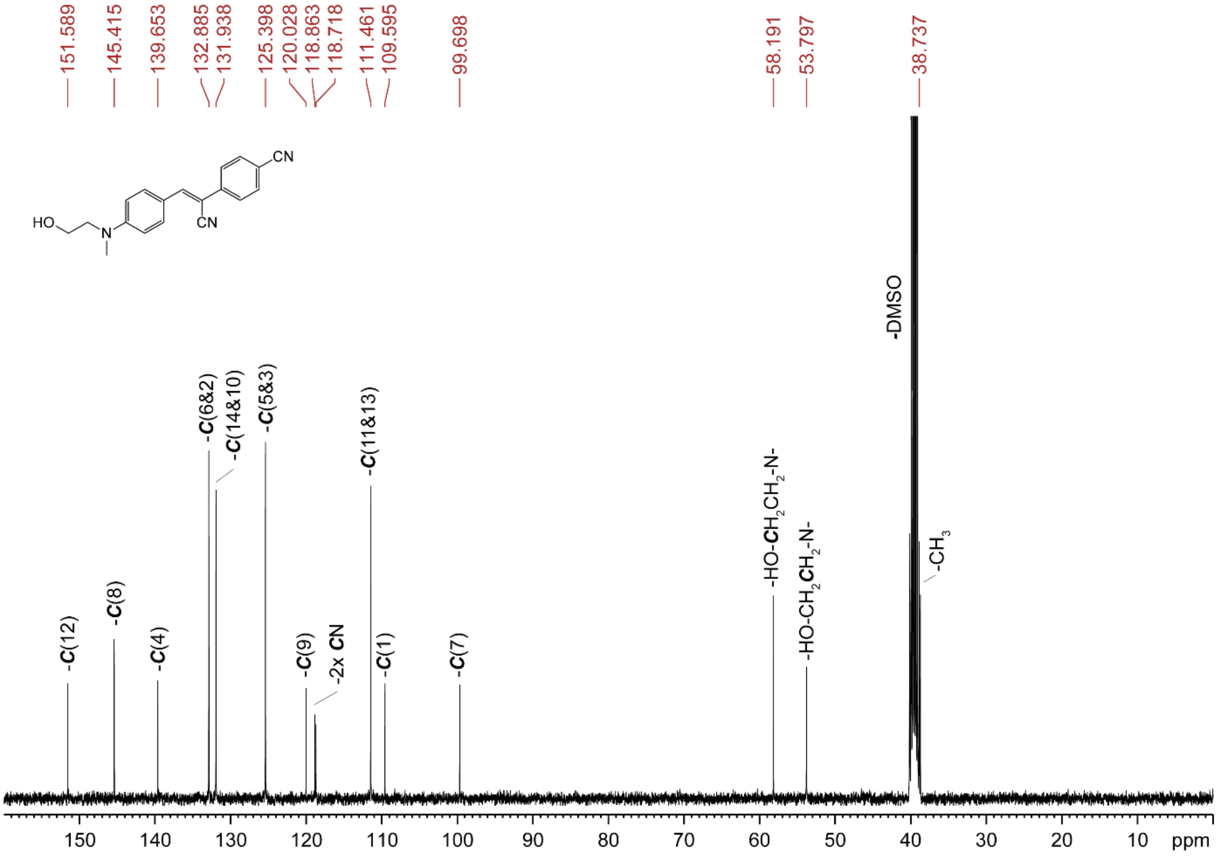

^{1}H NMR (400 MHz, DMSO-d_6, 25 °C) of compound **5**

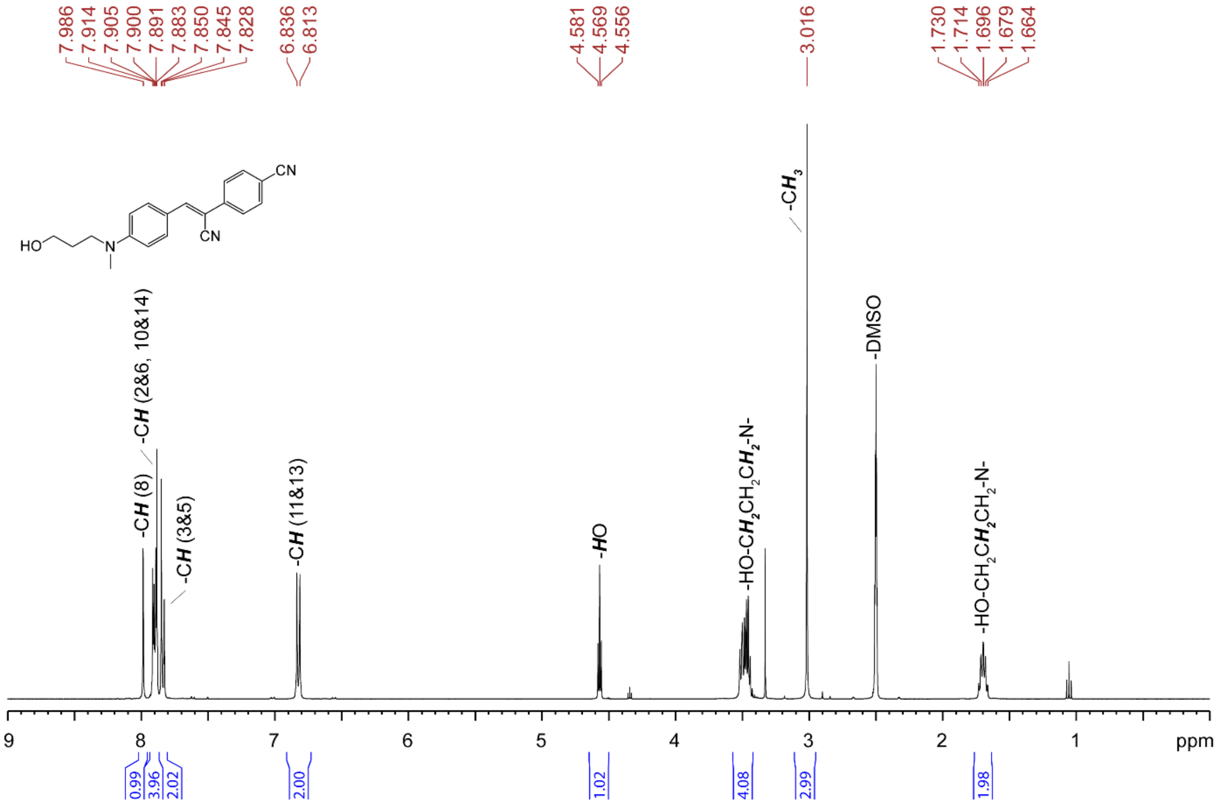

^{13}C NMR (100 MHz, DMSO-d_6, 25 °C) of compound **5**

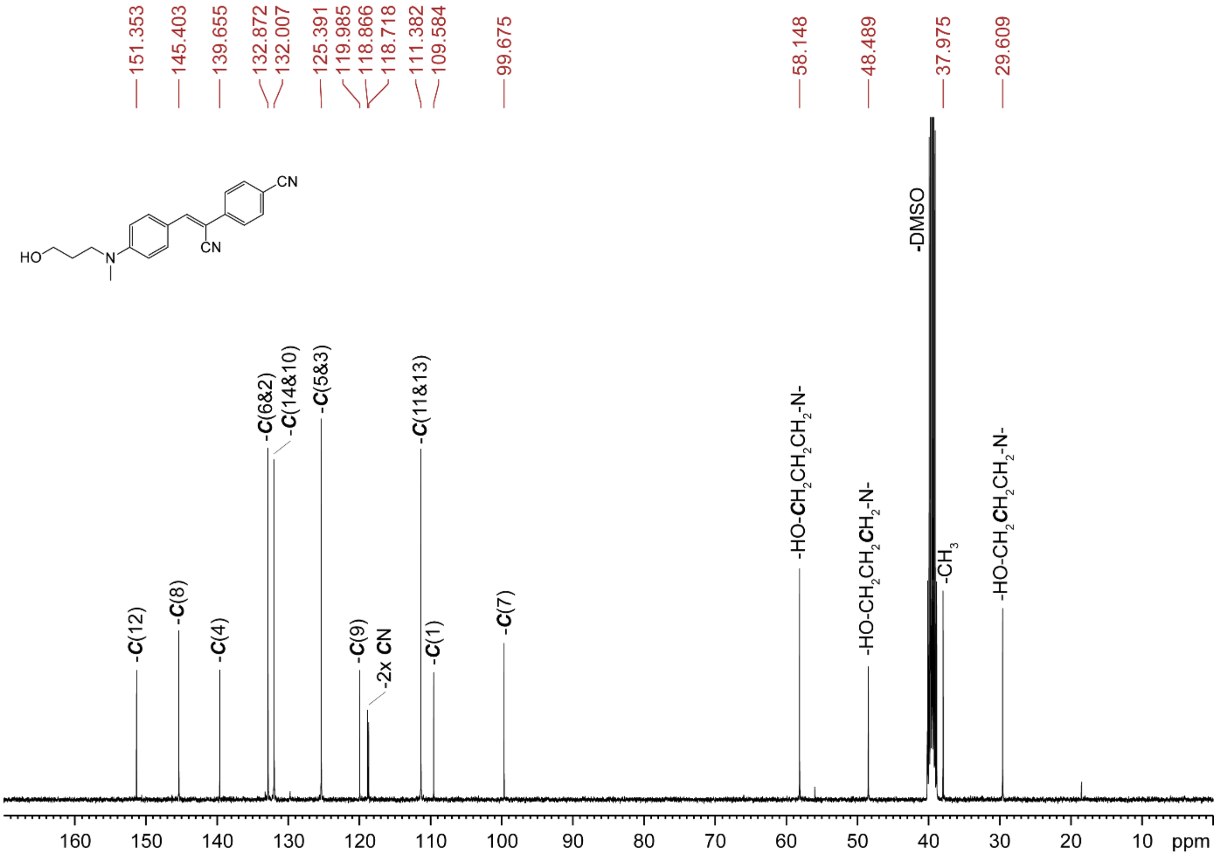

^{1}H NMR (400 MHz, DMSO-d_6, 25 °C) of compound **6**

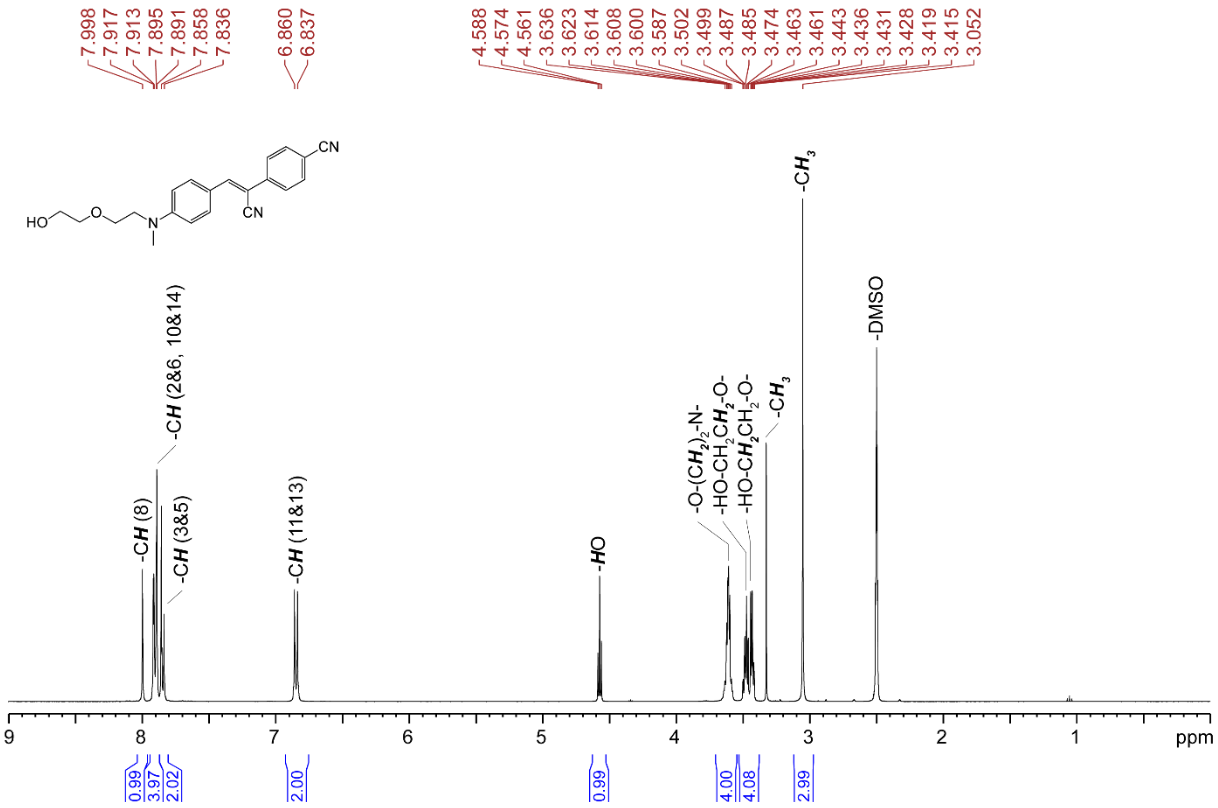

^{13}C NMR (100 MHz, DMSO-d_6, 25 °C) of compound **6**

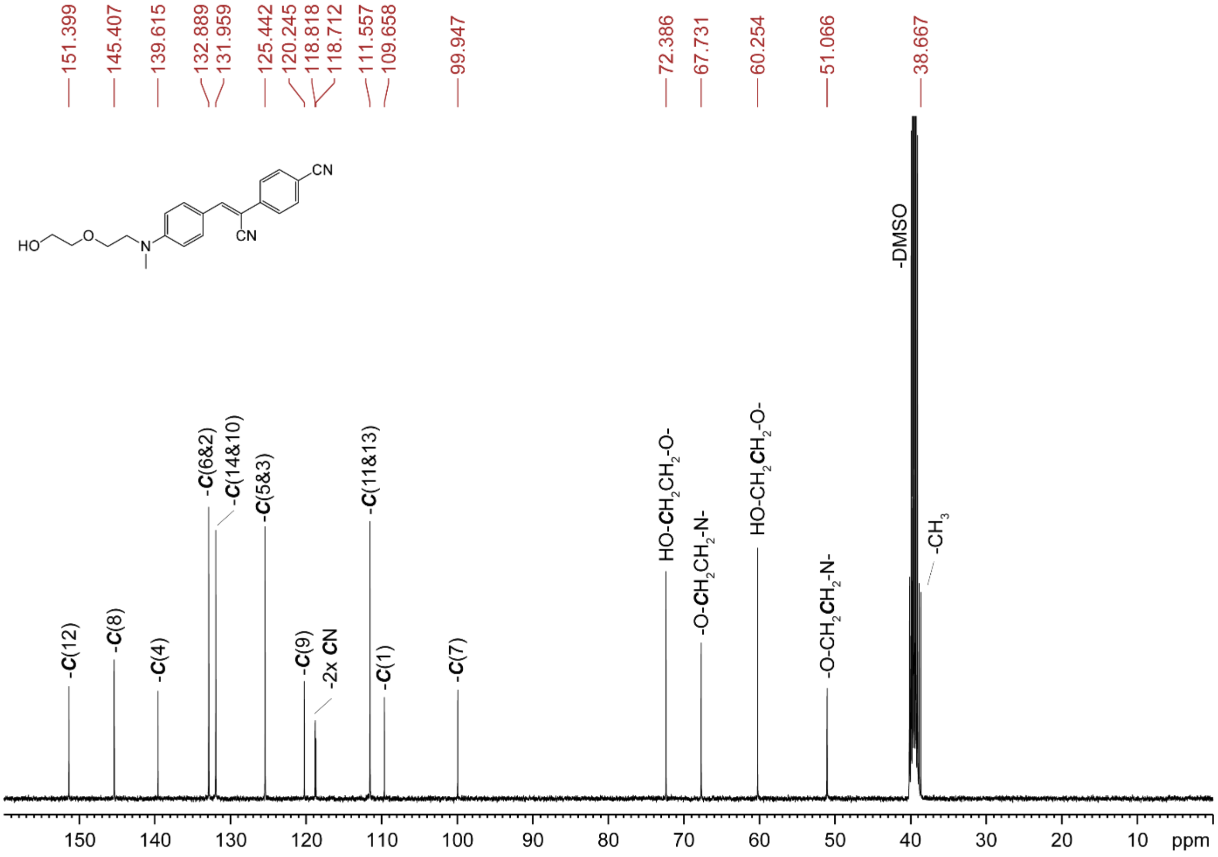

^{1}H NMR (400 MHz, DMSO-d_6, 25 °C) of compound **7**

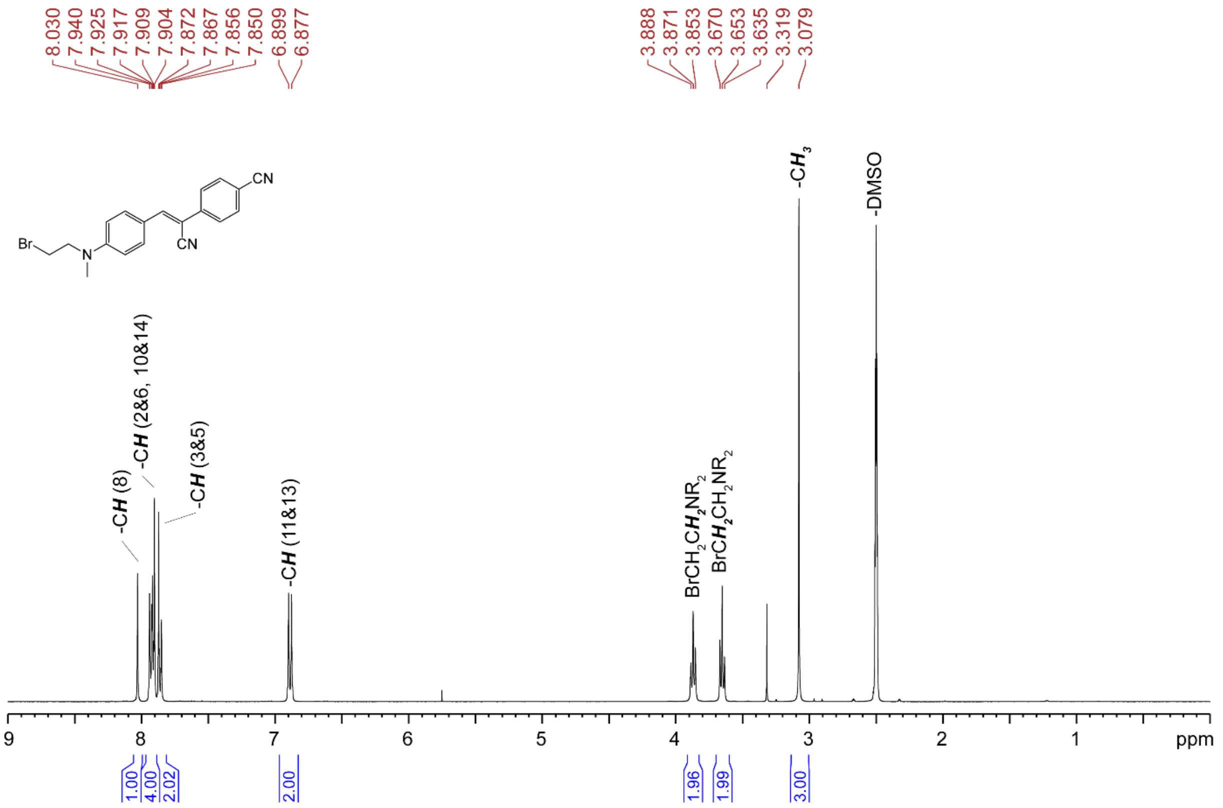

^{13}C NMR (100 MHz, DMSO-d_6, 25 °C) of compound **7**

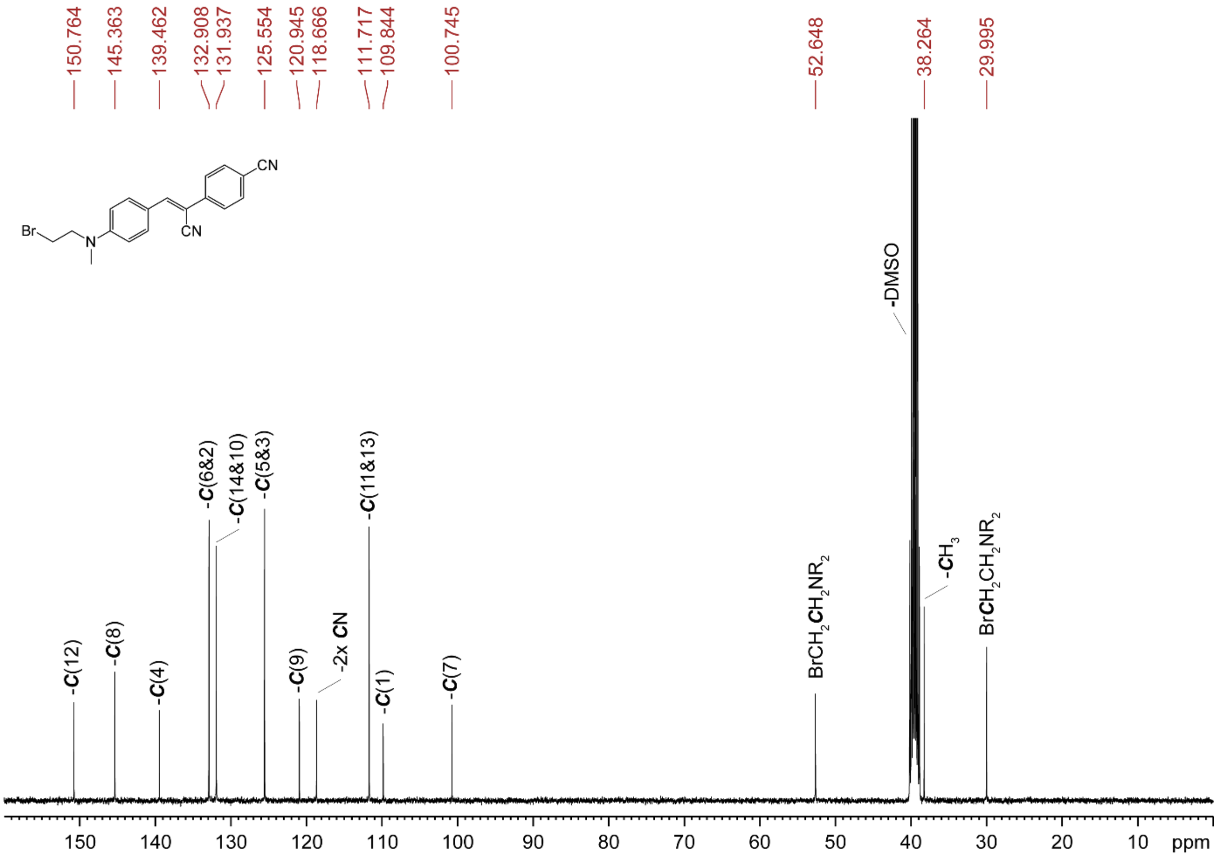

^{1}H NMR (400 MHz, DMSO-d_6, 25 °C) of compound **9**

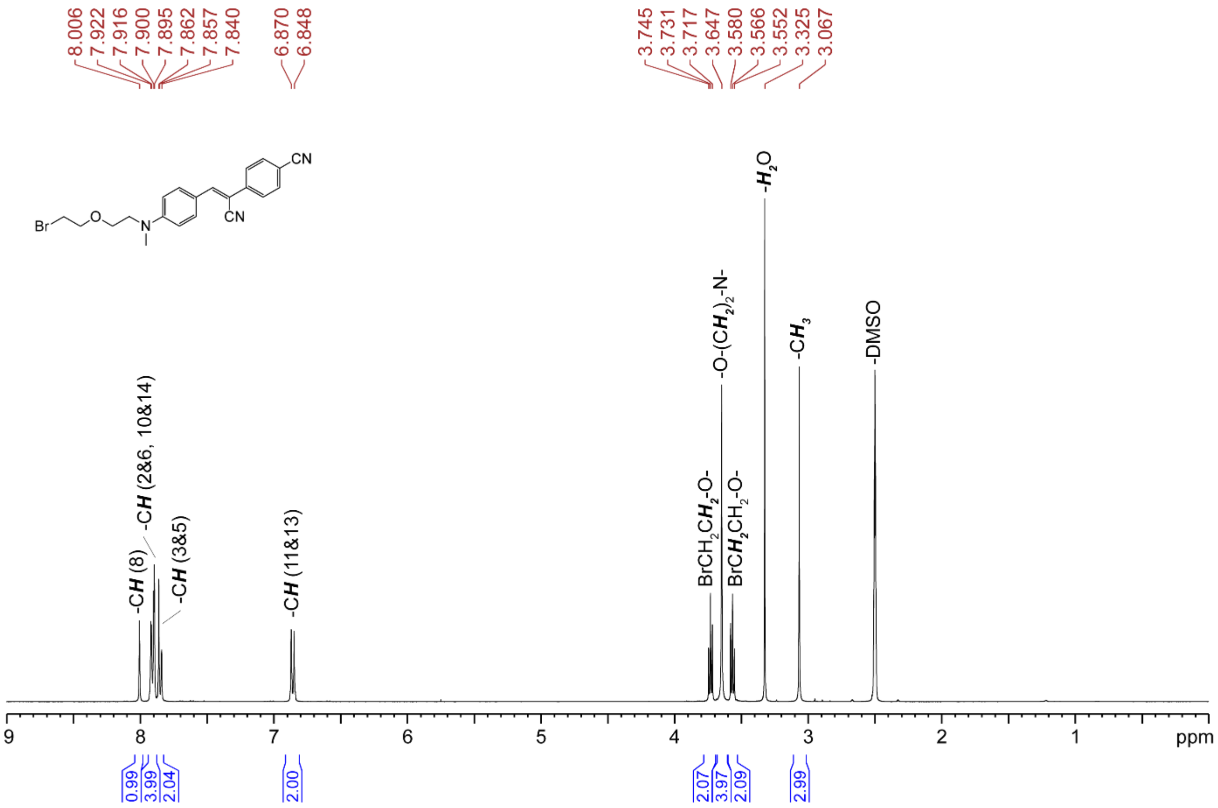

^{13}C NMR (100 MHz, DMSO-d_6, 25 °C) of compound **9**

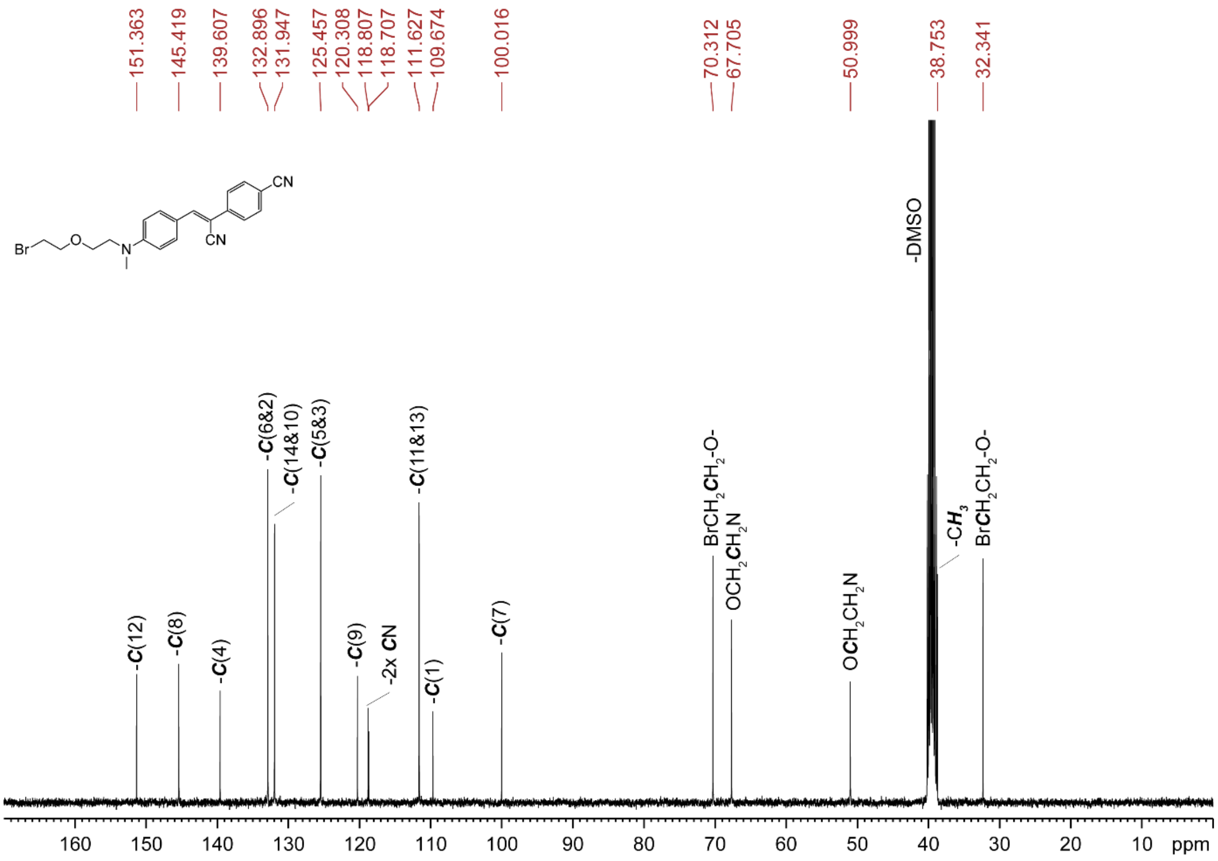

^{1}H NMR (400 MHz, DMSO-d_6, 25 °C) of compound **10**

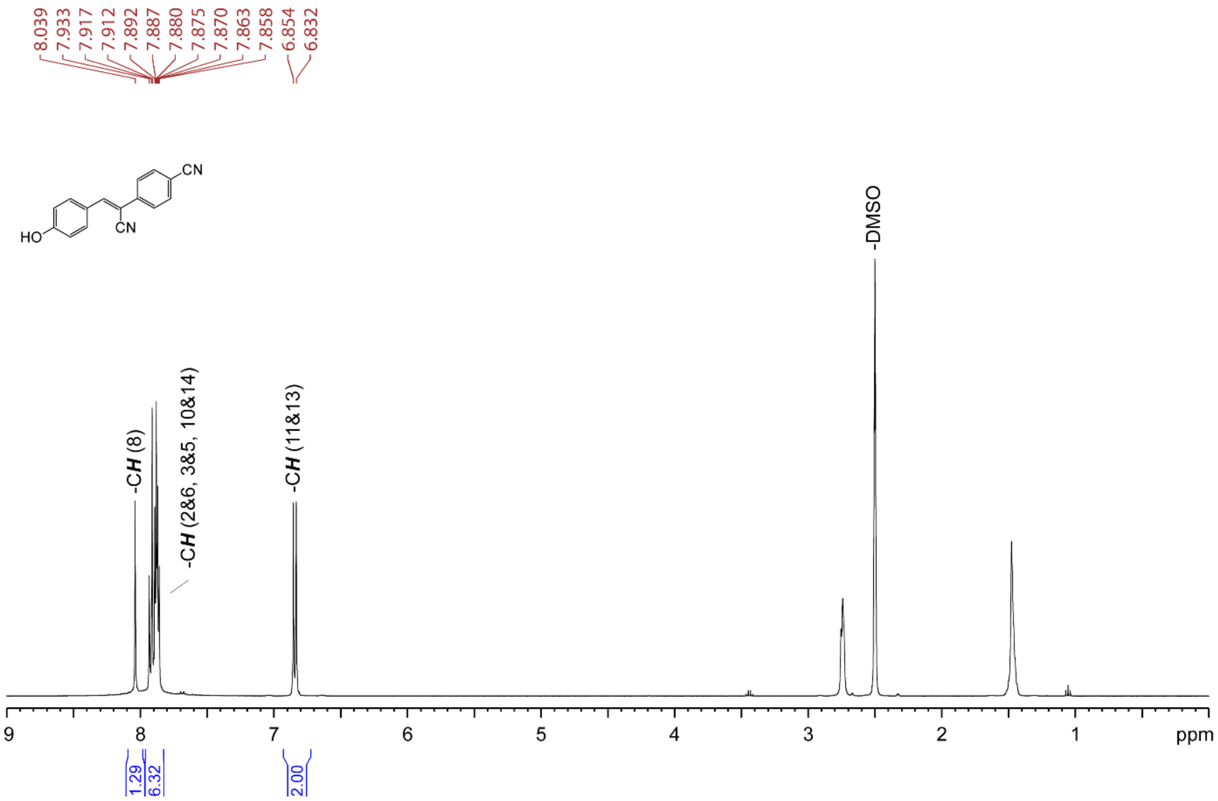

^{13}C NMR (100 MHz, DMSO-d_6, 25 °C) of compound **10**

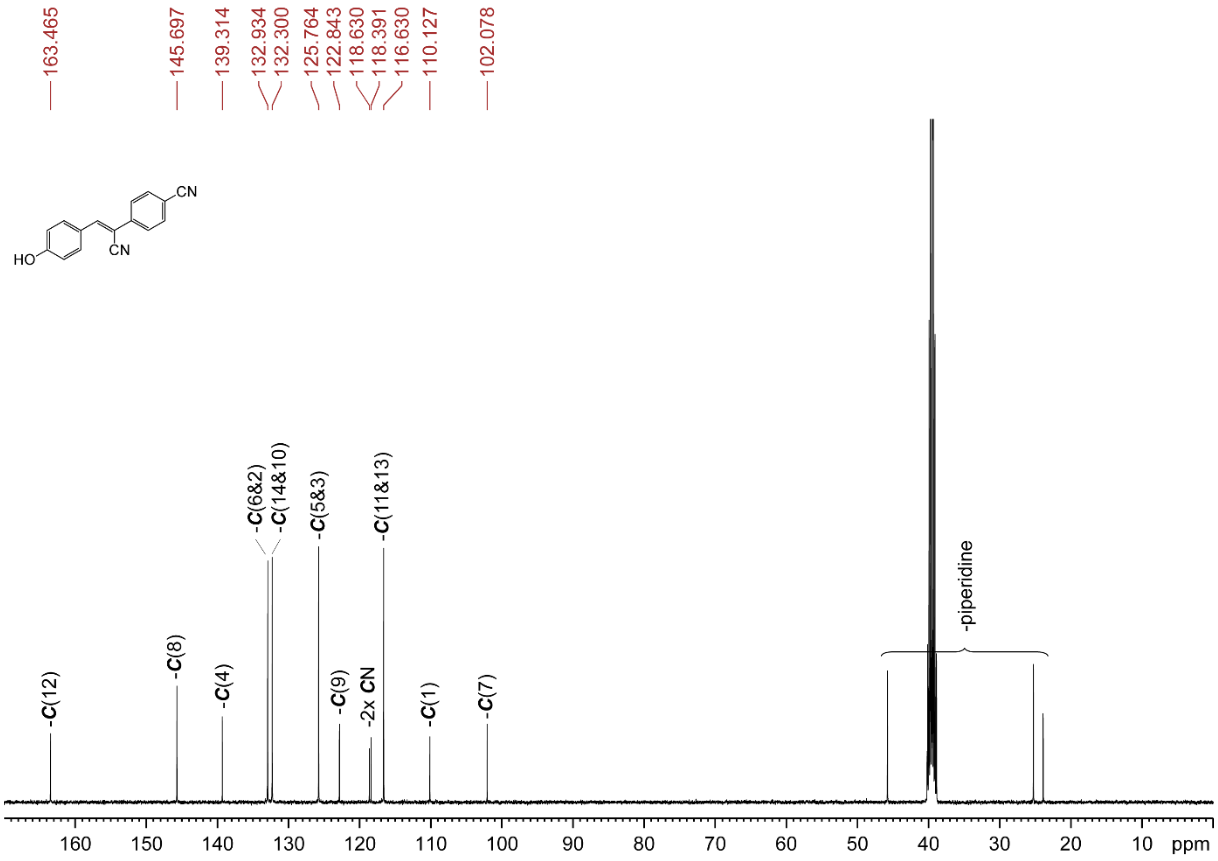

^{1}H NMR (400 MHz, DMSO-d_6, 25 °C) of compound **11**

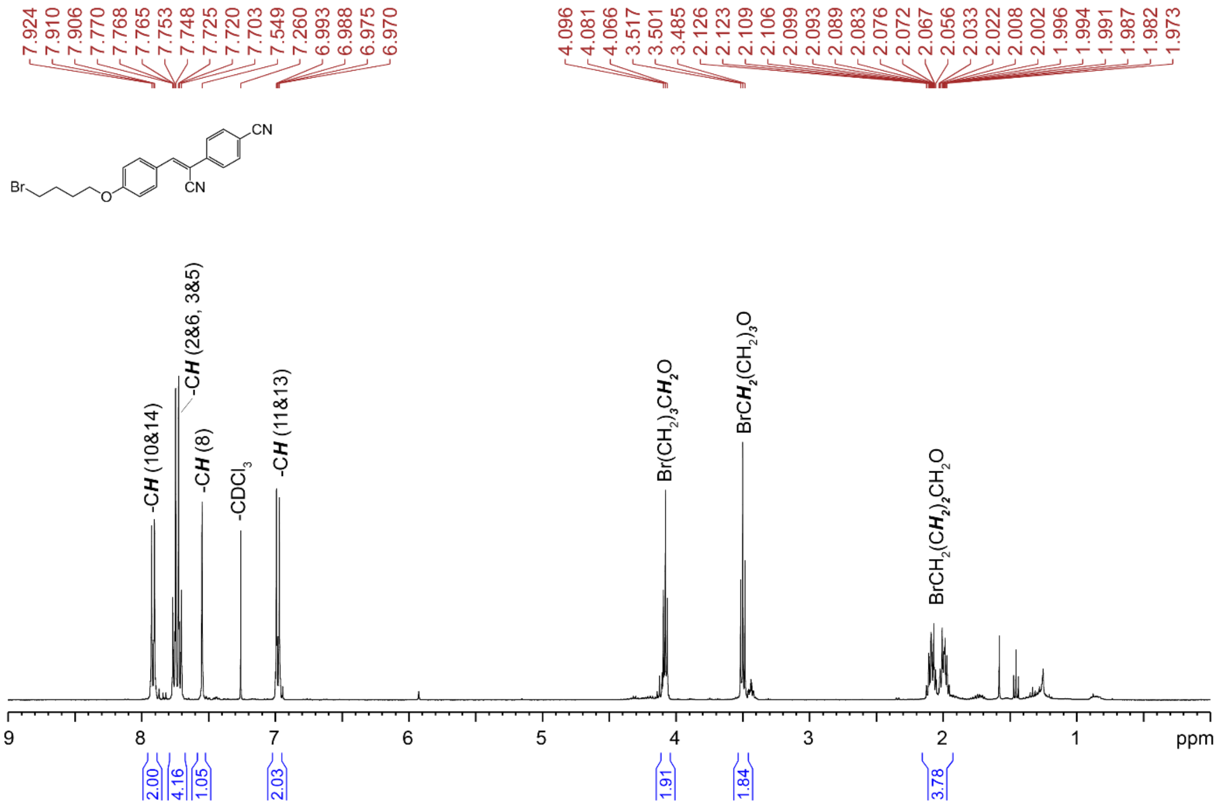

^{13}C NMR (100 MHz, DMSO-d_6, 25 °C) of compound **11**

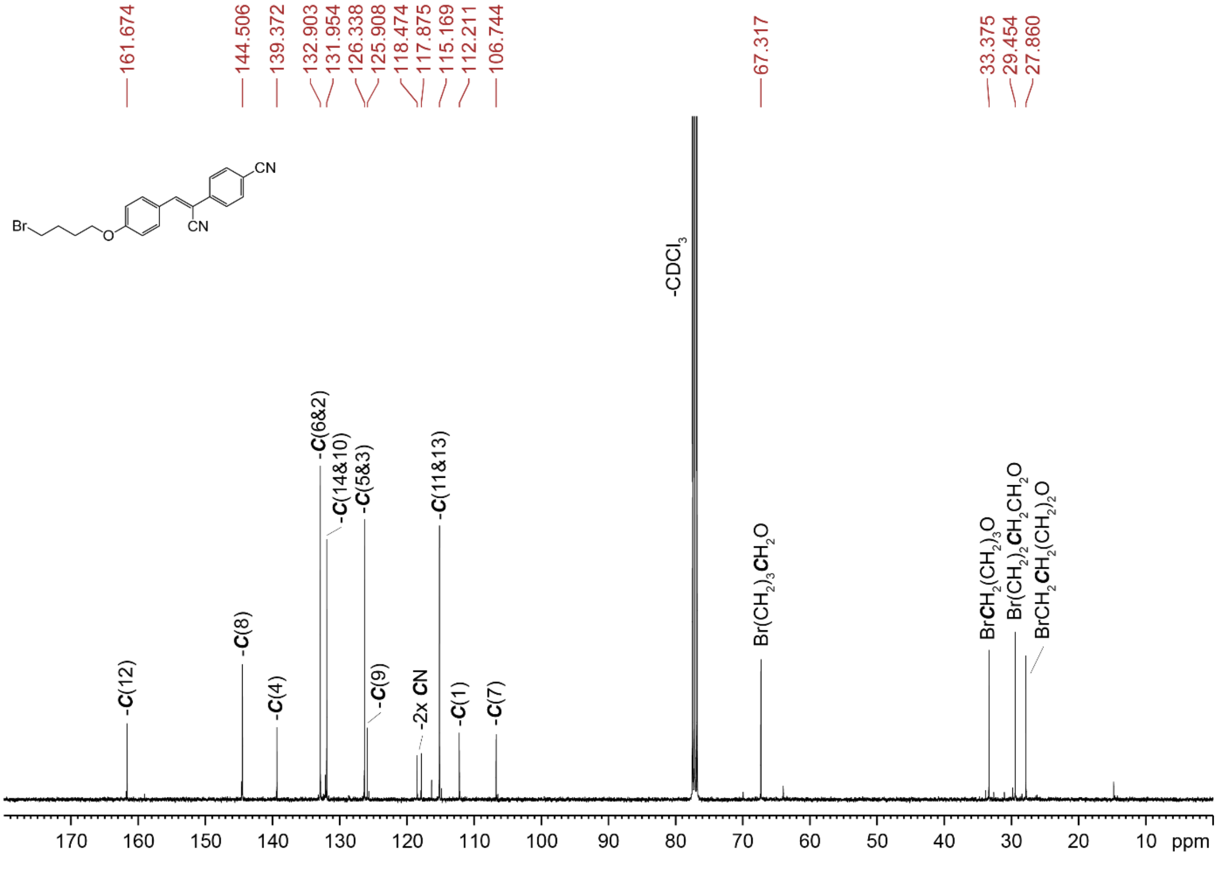

^{1}H NMR (400 MHz, $CDCl_3$, 25 °C) of compound **12**

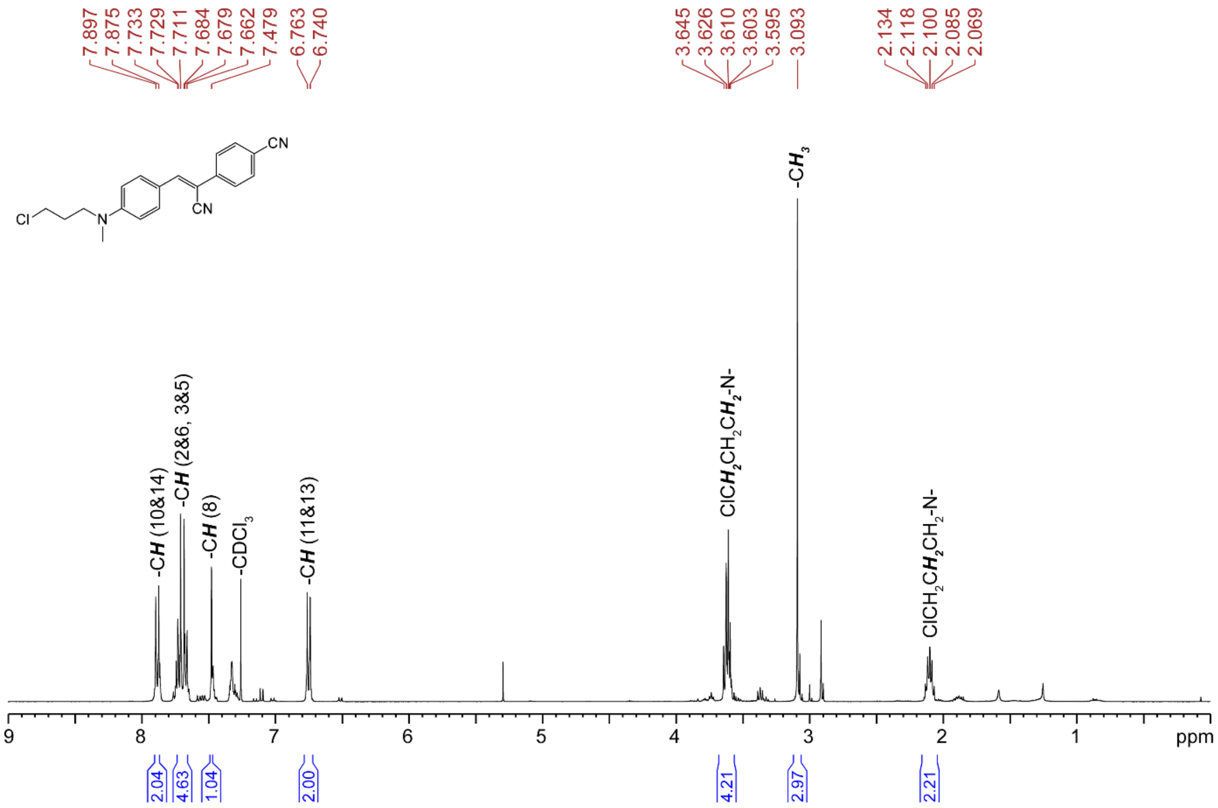

^{13}C NMR (100 MHz, $CDCl_3$, 25 °C) of compound **12**

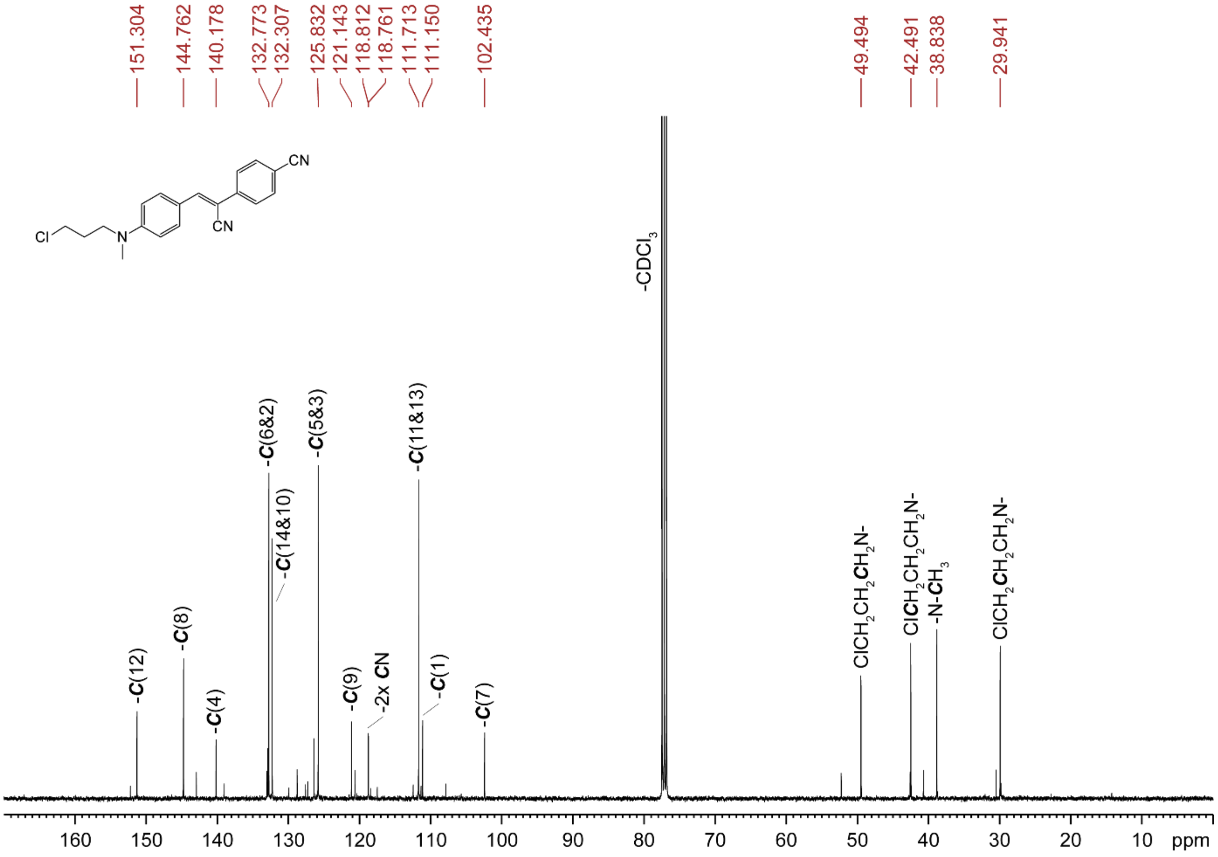

^{1}H NMR (400 MHz, $CDCl_3$, 25 °C) of compound **13**

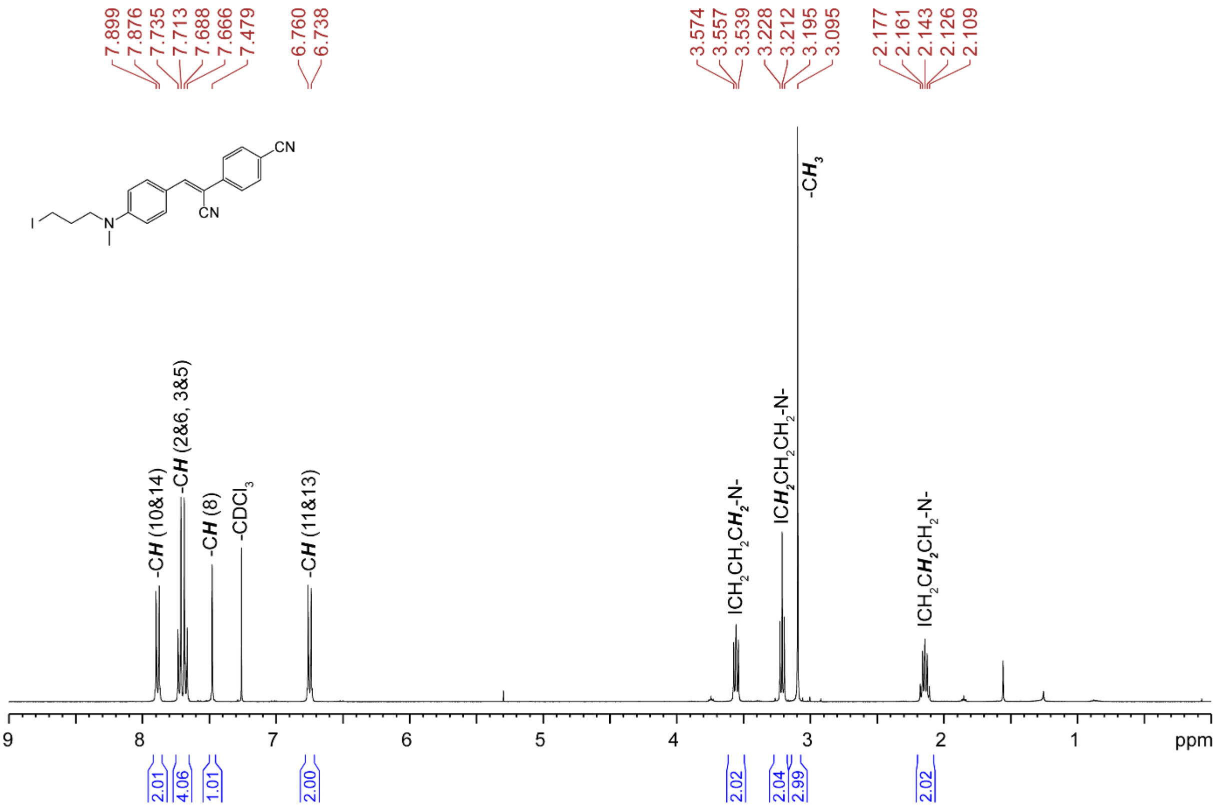

^{13}C NMR (100 MHz, $CDCl_3$, 25 °C) of compound **13**

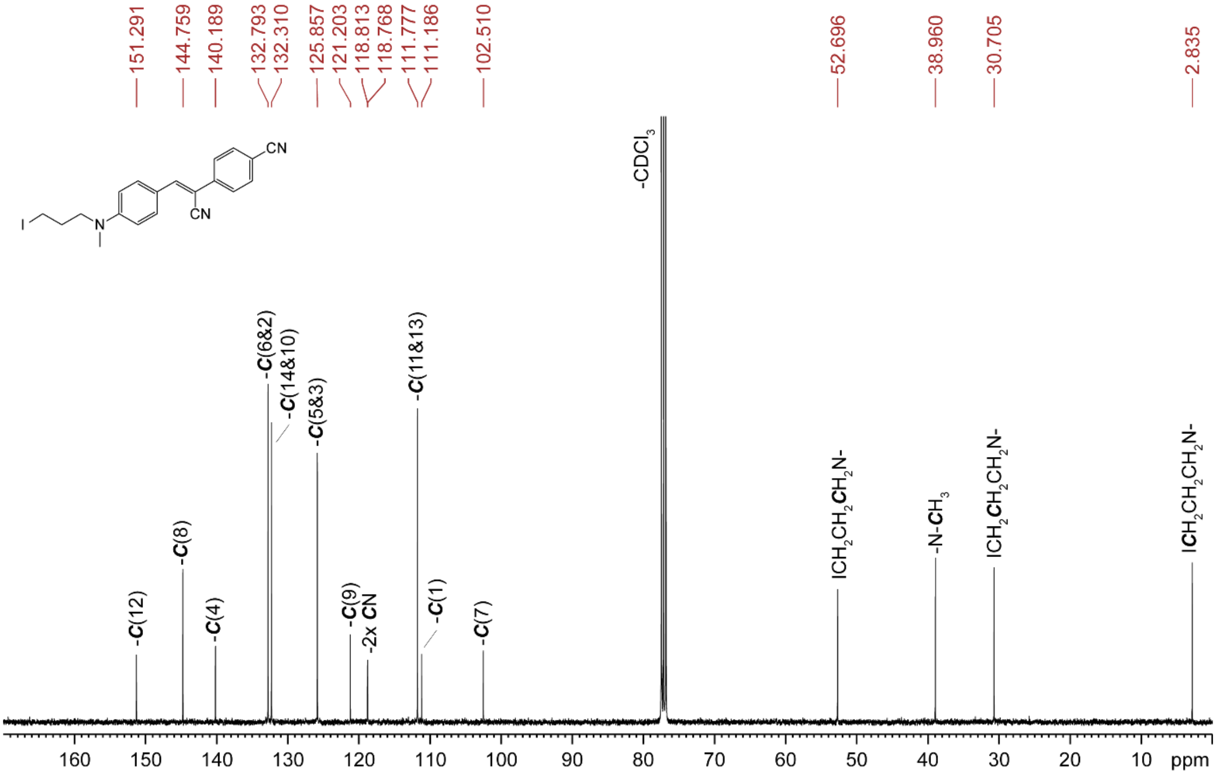

^{1}H NMR (400 MHz, $CDCl_3$, 25 °C) of compound **15**

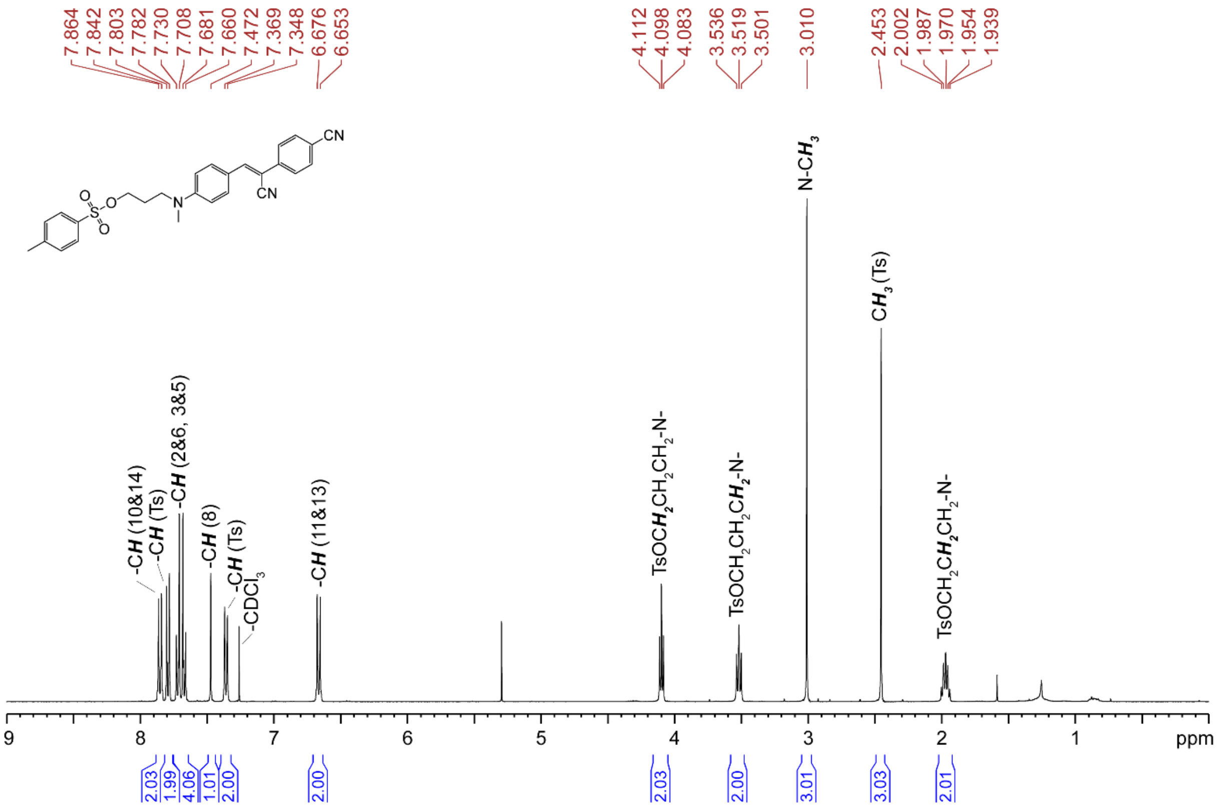

^{13}C NMR (100 MHz, $CDCl_3$, 25 °C) of compound **15**

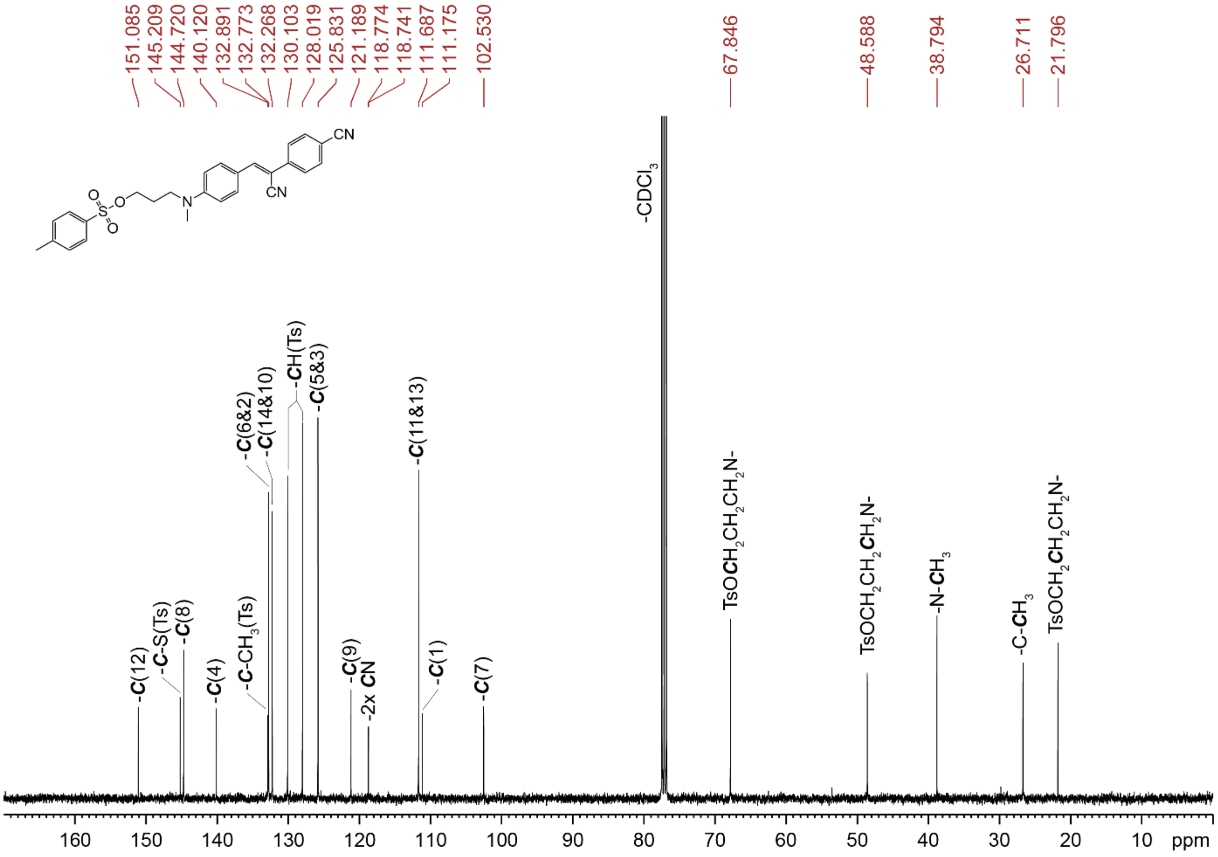

^{1}H NMR (400 MHz, $CDCl_3$, 25 °C) of compound **16**

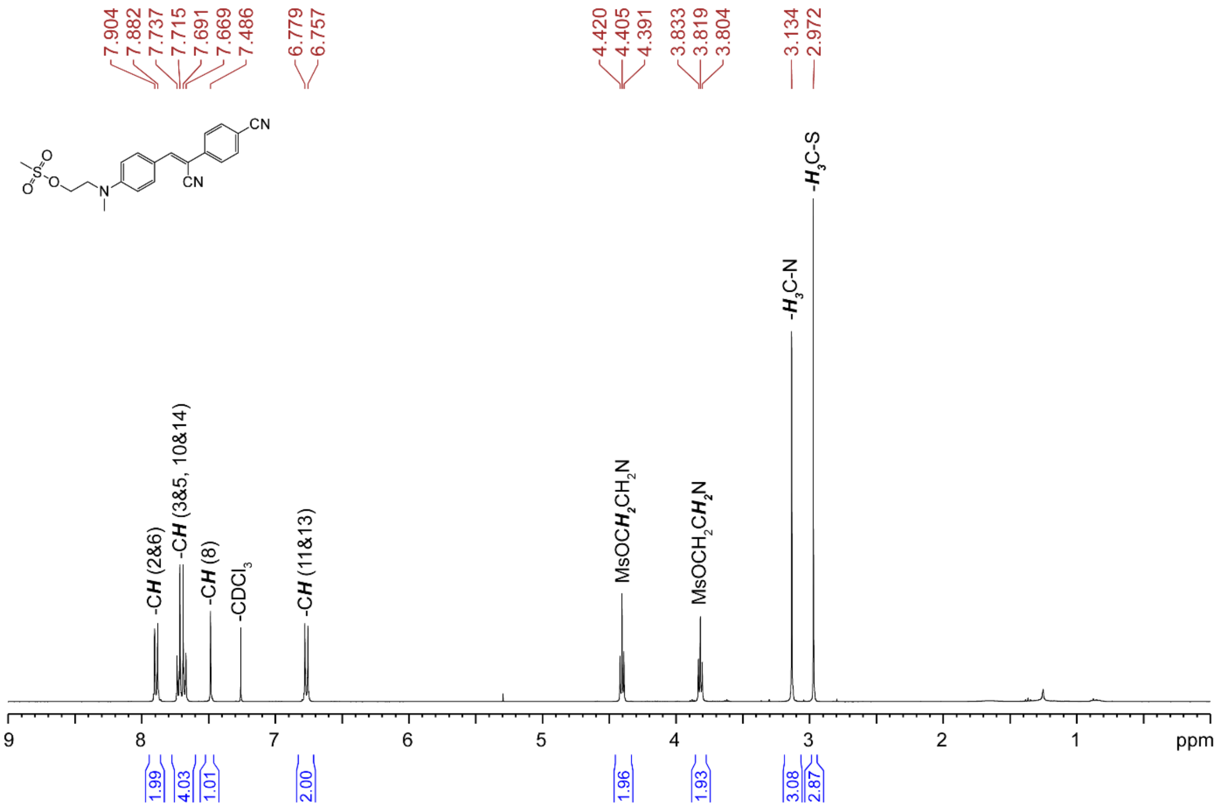

^{13}C NMR (100 MHz, $CDCl_3$, 25 °C) of compound **16**

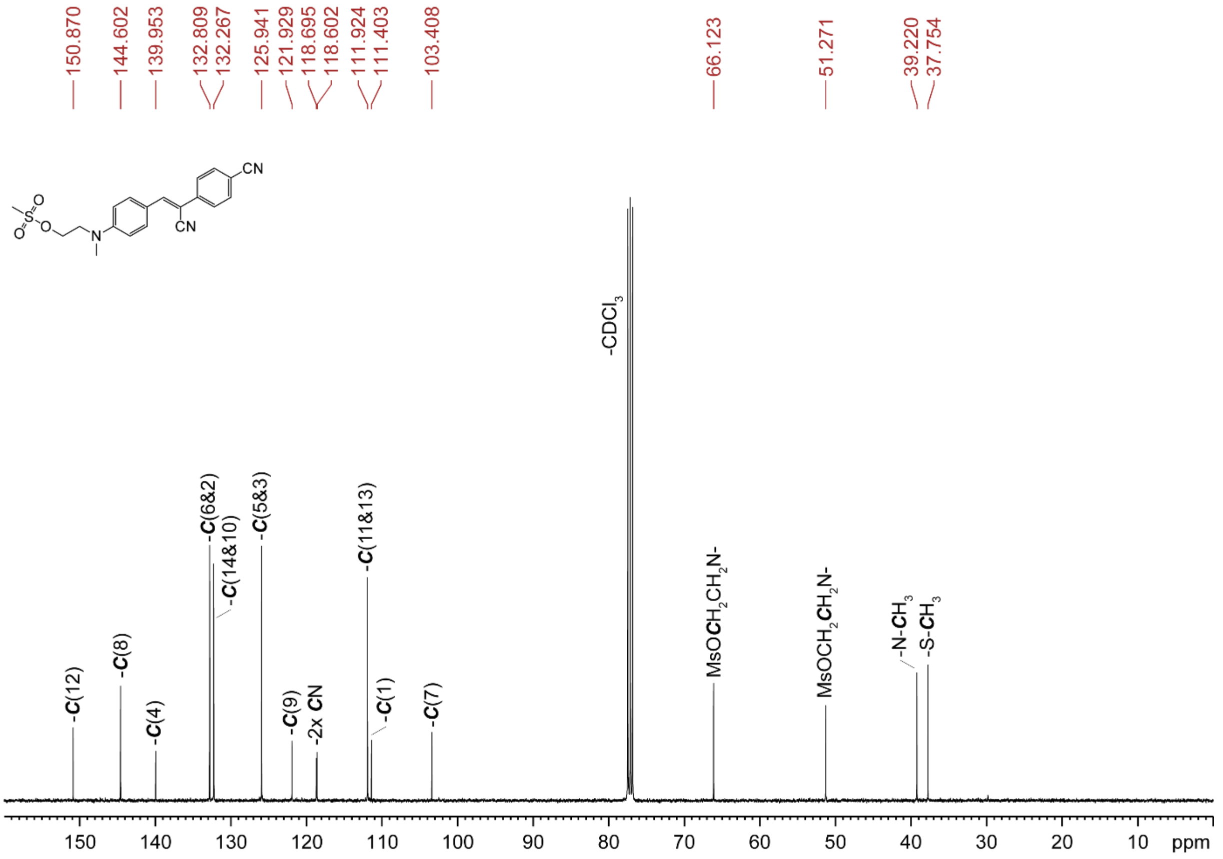

^{1}H NMR (400 MHz, $CDCl_3$, 25 °C) of compound **17**

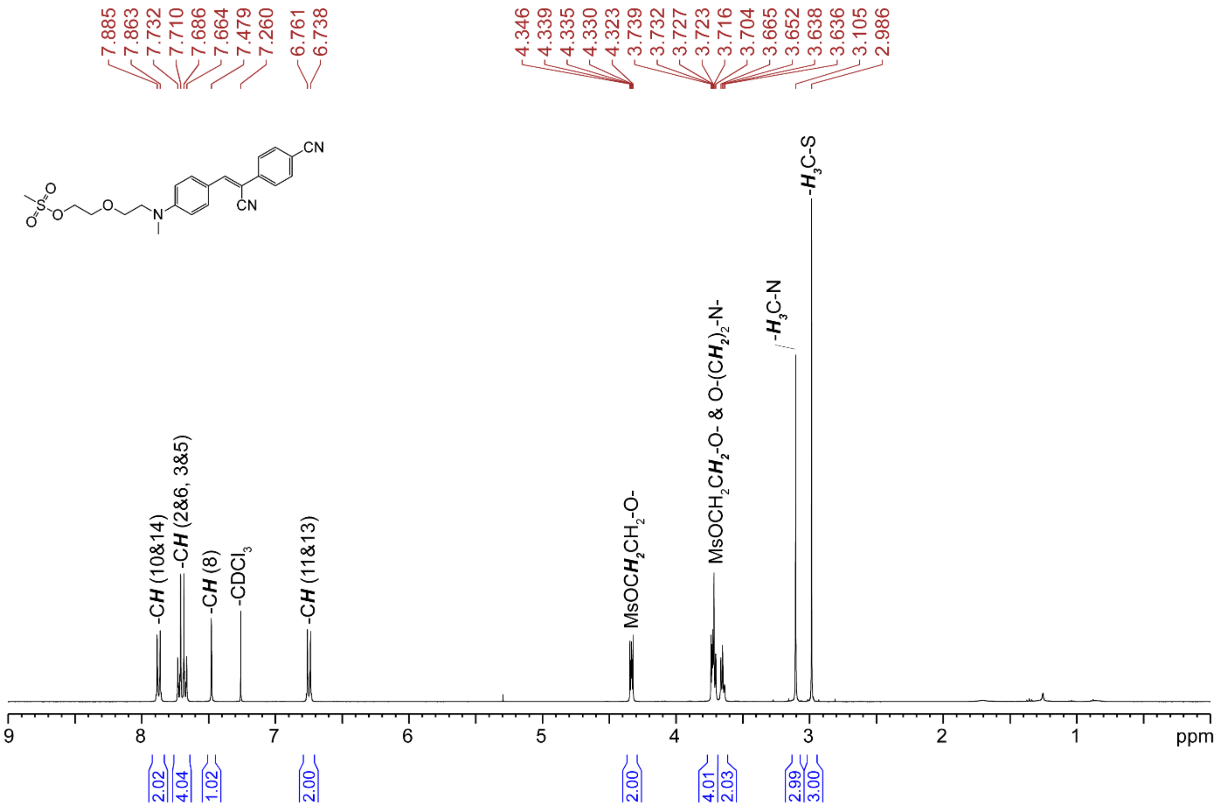

^{13}C NMR (100 MHz, $CDCl_3$, 25 °C) of compound **17**

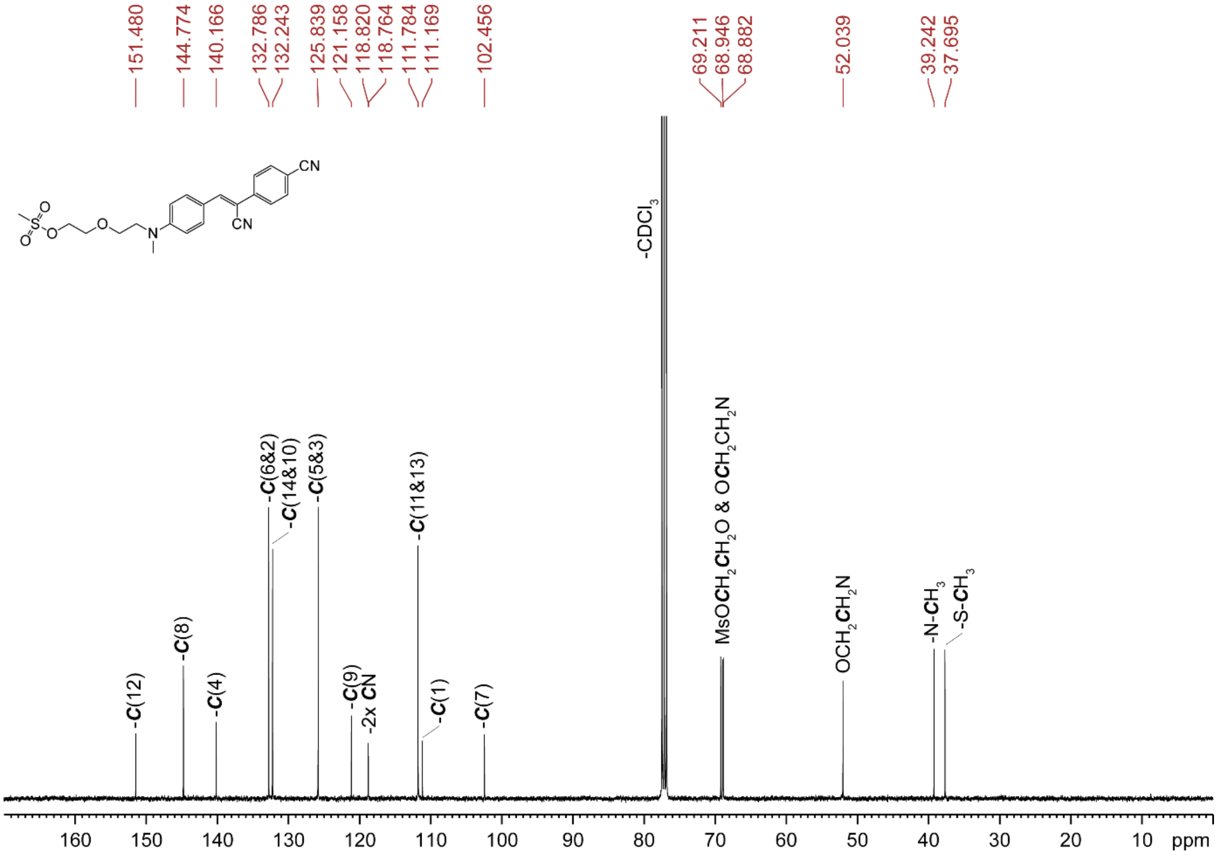

^1H NMR (400 MHz, $CDCl_3$, 25 °C) of compound **18**

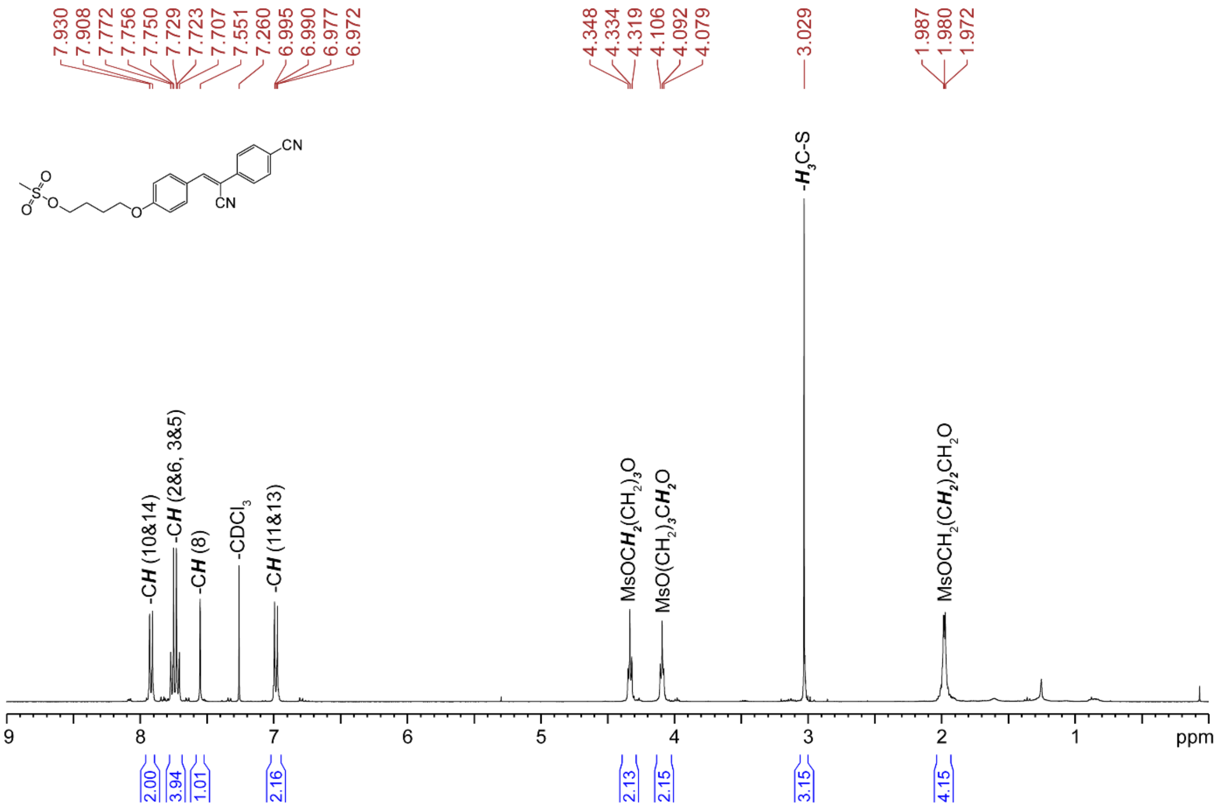

^{13}C NMR (100 MHz, $CDCl_3$, 25 °C) of compound **18**

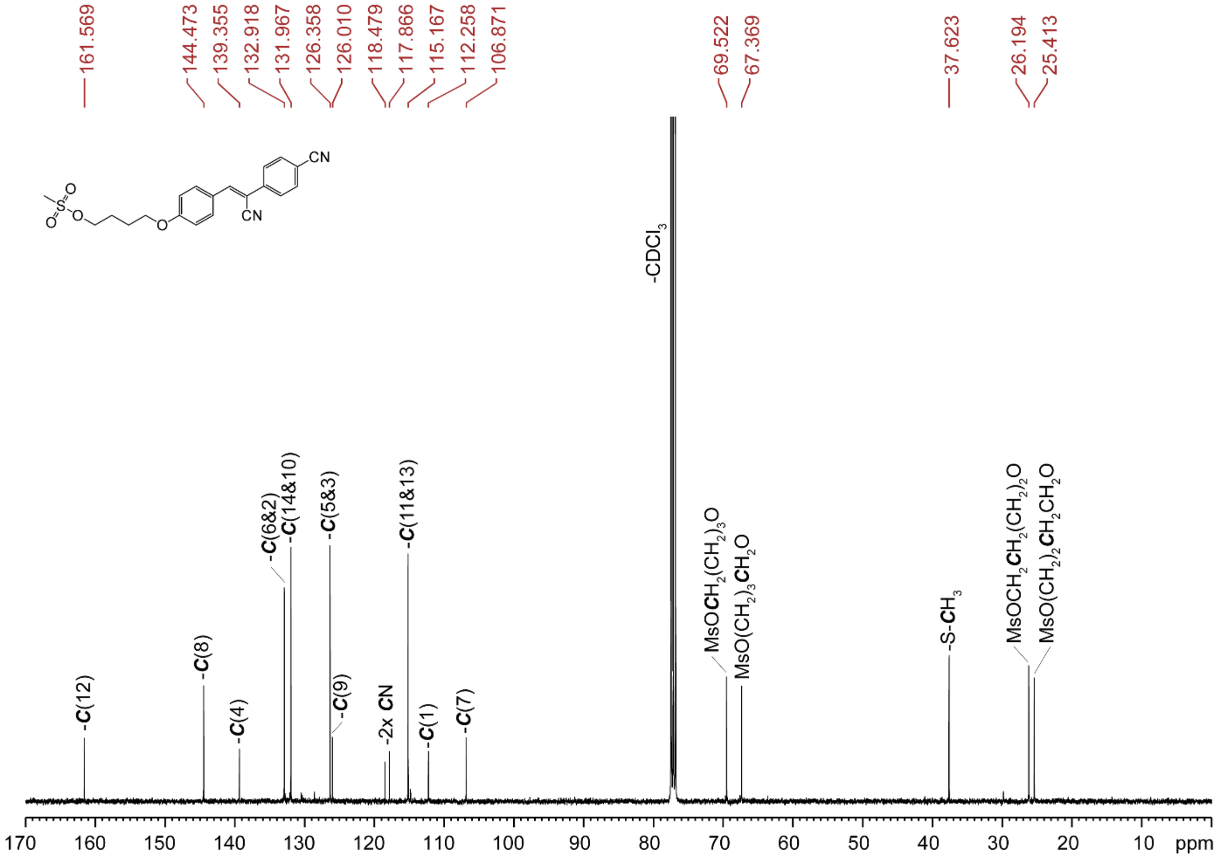

^{1}H NMR (400 MHz, $CDCl_3$, 25 °C) of compound **19**

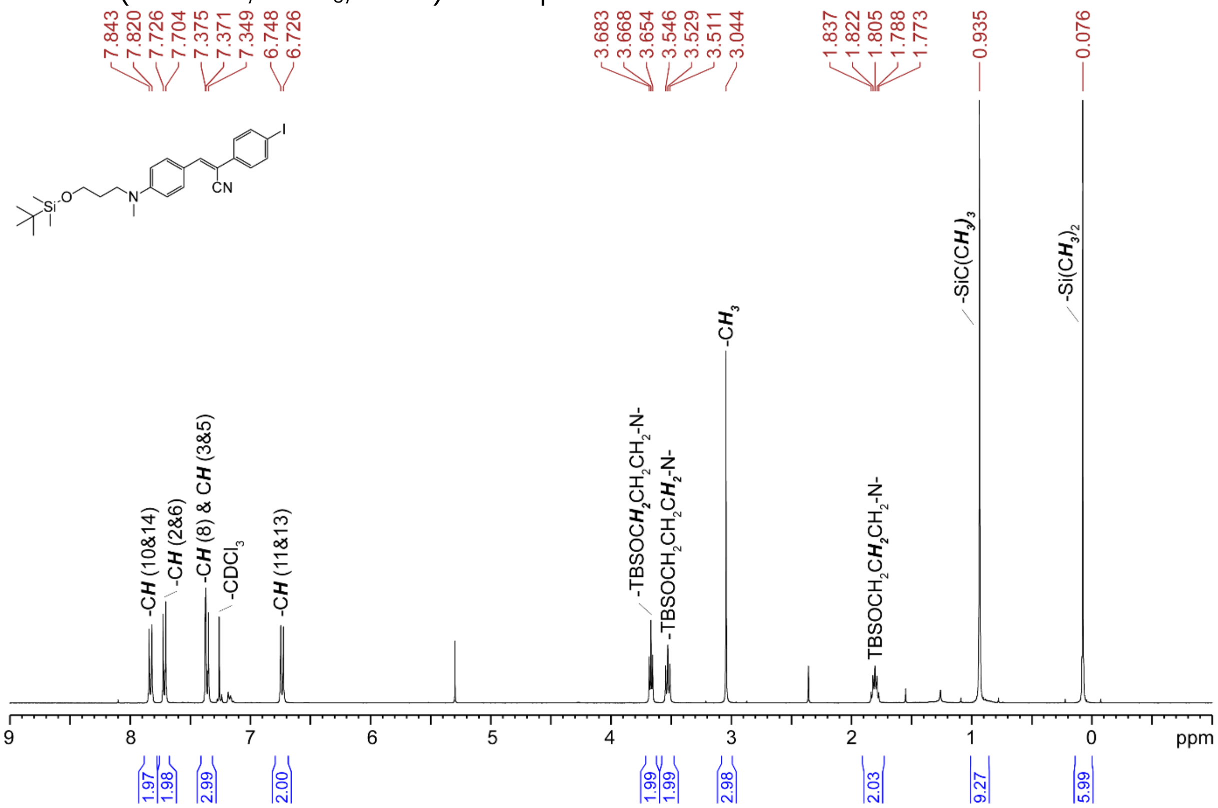

^{13}C NMR (100 MHz, $CDCl_3$, 25 °C) of compound **19**

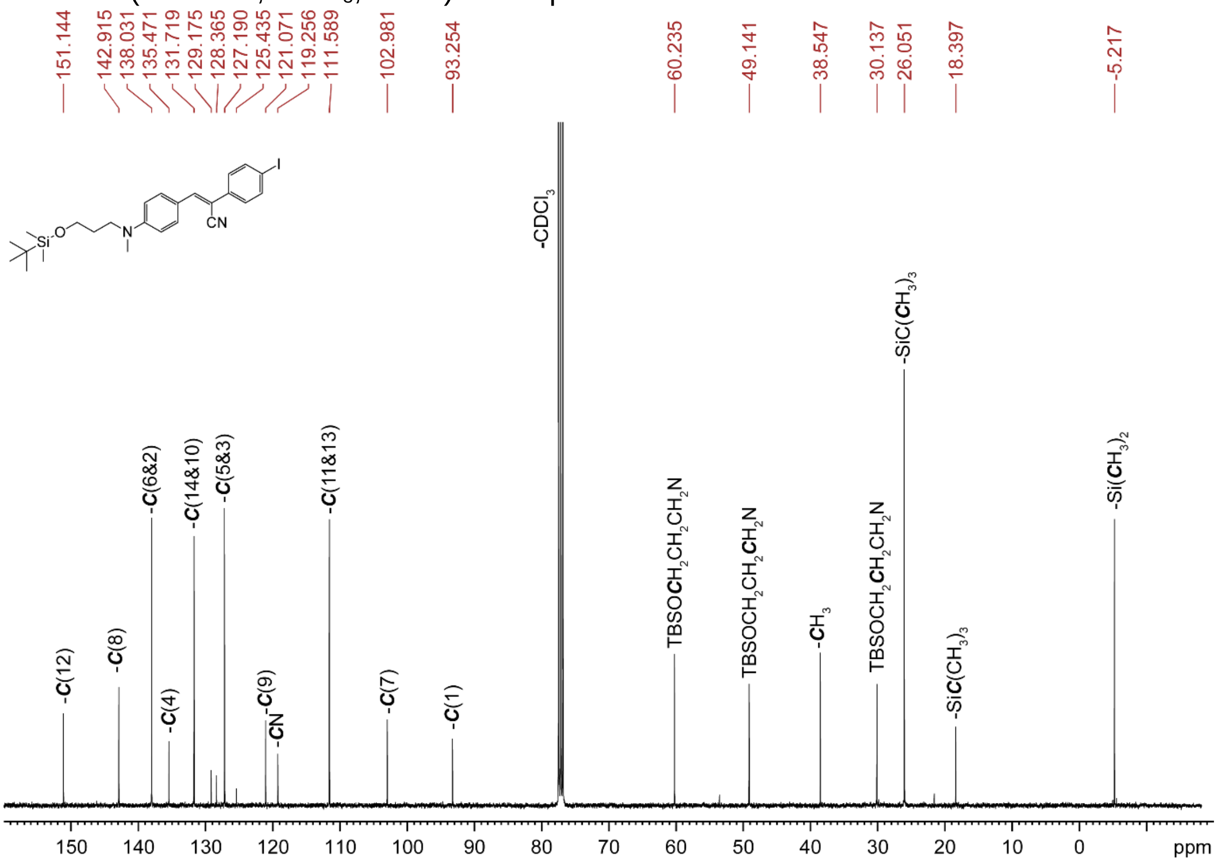

^{1}H NMR (400 MHz, $CDCl_3$, 25 °C) of compound **20**

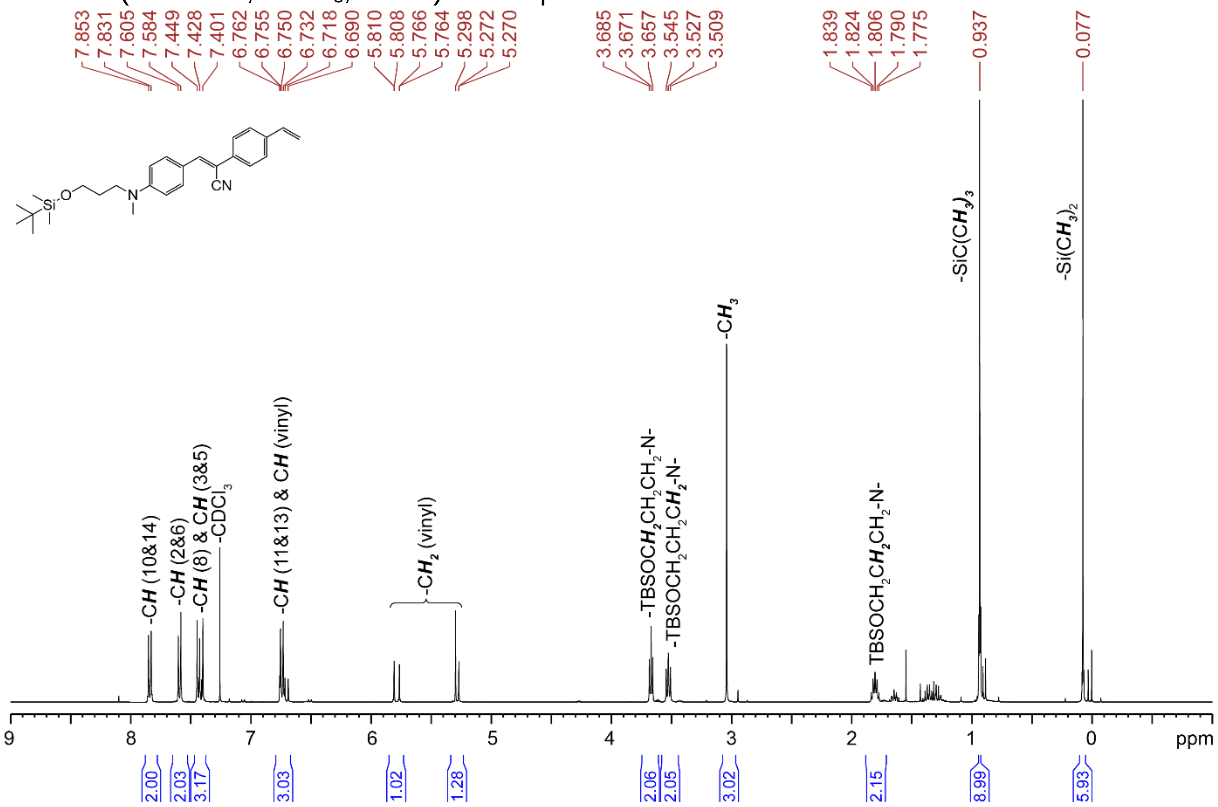

^{13}C NMR (100 MHz, $CDCl_3$, 25 °C) of compound **20**

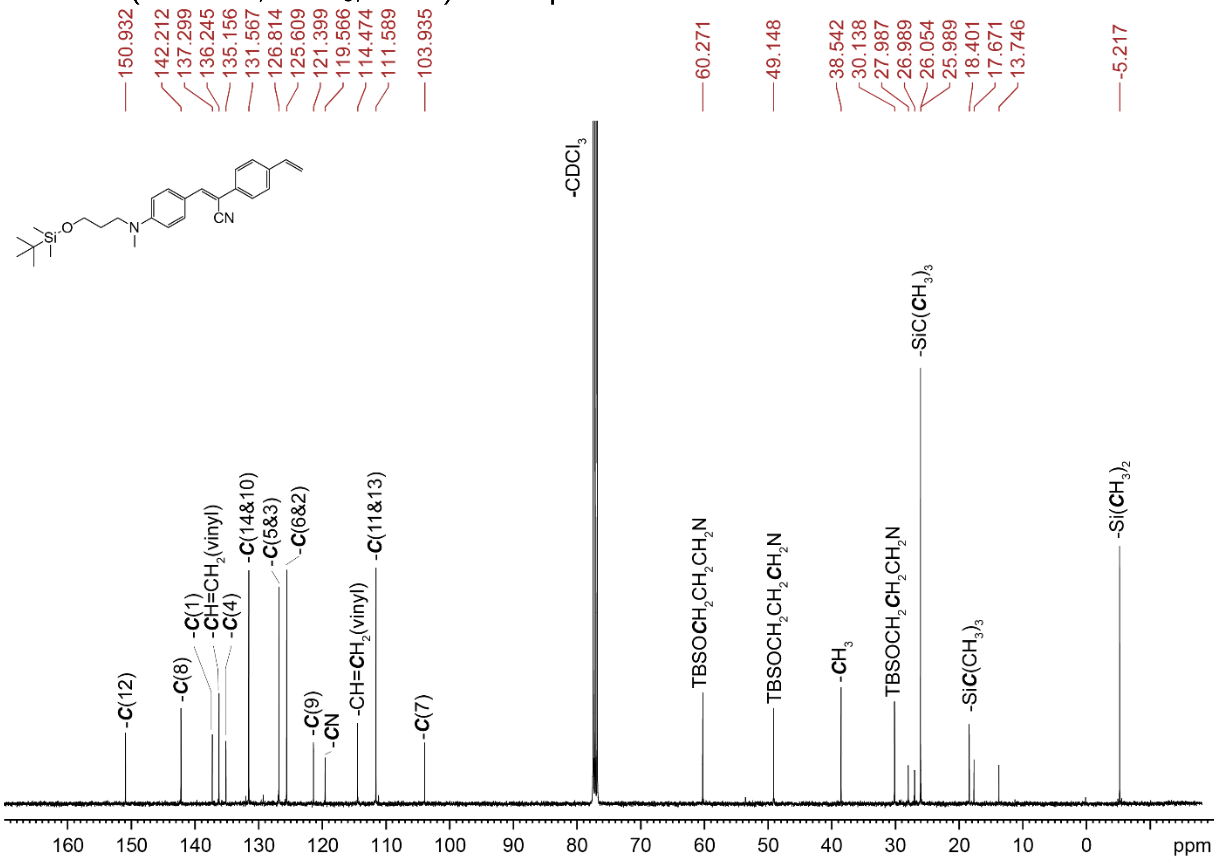

^{1}H NMR (400 MHz, DMSO-*d*6, 25 °C) of compound **21**

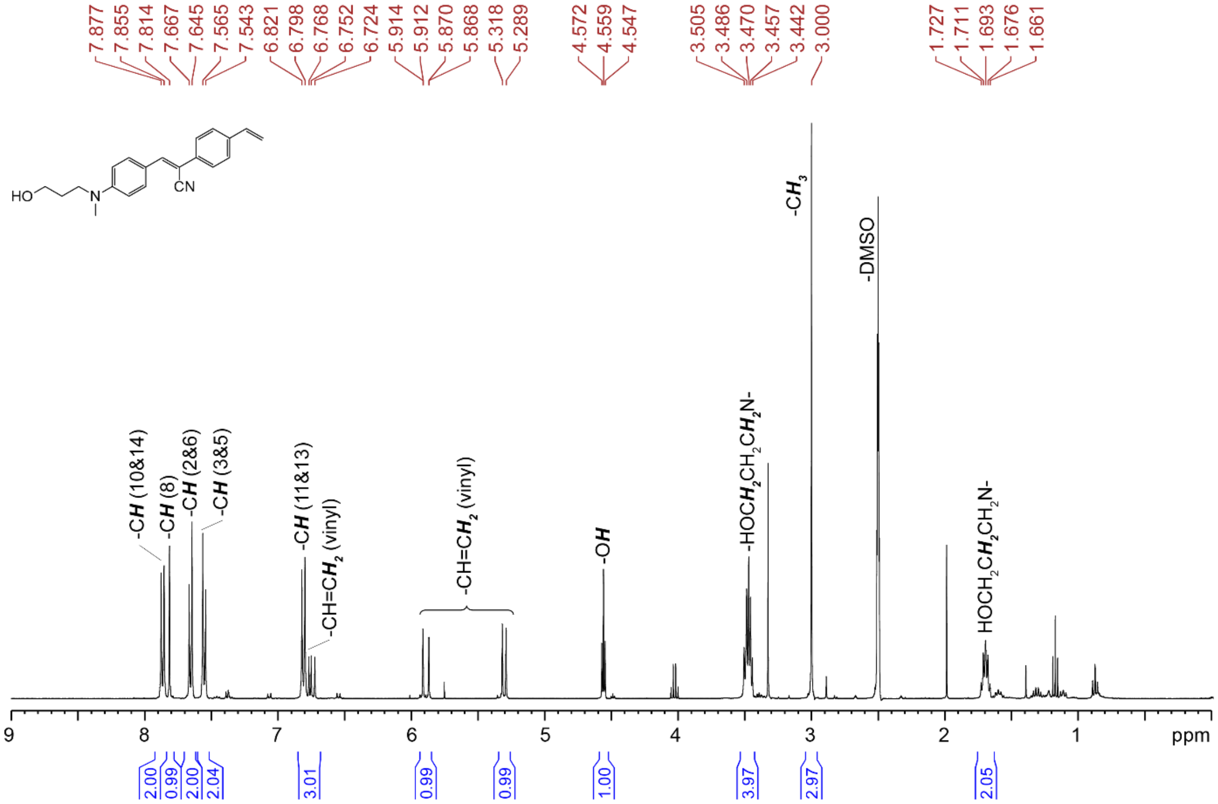

^{13}C NMR (100 MHz, DMSO-*d*6, 25 °C) of compound **21**

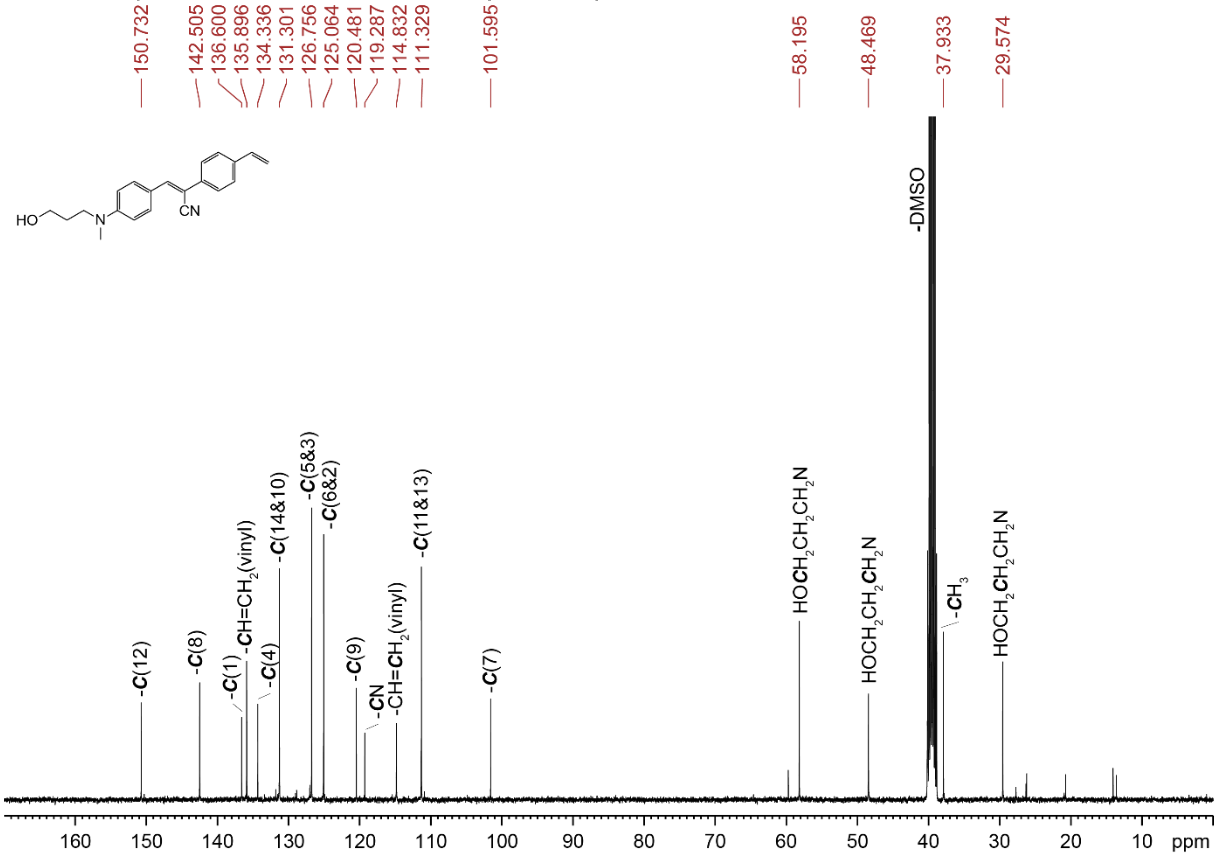

Supplement: File 1 — Experimental, characterization data and copies of NMR spectra. [file Beilstein_J_Org_Chem-21-727-s001.pdf]
